# Supplementary material for: Selective far-field addressing of coupled quantum dots in a plasmonic nanocavity
Source: Nat Commun. 2018 Apr 27;9:1705. doi: 10.1038/s41467-018-04077-z (PMC5924364; doi:10.1038/s41467-018-04077-z)
Supplement: Supplementary file 1 — Supplementary Information [file 41467_2018_4077_MOESM1_ESM.pdf]

Supplementary Information for

Selective far-field addressing of coupled quantum dots in a plasmonic nanocavity

Tang *et al.*

List of contents

Supplementary Note 1 - Structure parameters of the nanosystem

Supplementary Note 2 - Mechanism for selective excitation

Supplementary Note 3 - Purcell effect for QDs in the plasmonic nanostructure

Supplementary Note 4 - Energy transfer between the QDs

Supplementary Note 5 - Finding optimal polarization for selective excitation and optimal polarizer angle for selective detection

Supplementary Note 6 - Exponential fitting of fluorescence decay curves

Supplementary Note 7 - Photon collection efficiency

Supplementary Note 8 - Plasmon-mediated entanglement

Supplementary Figure 1 to **16**

Supplementary Table 1

Supplementary References

Supplementary Note 1 - Structure parameters of the nanosystem

The structure parameters (see Supplementary Fig. 3 for the definitions of the parameters) of the experimentally reported nanosystem (Fig. 1b) are estimated as follows. The diameters of the GNRs and the silica-encapsulated QDs are estimated according to AFM topography images. The diameters of G1, G2 and G3 are estimated to be 24.8 nm (d_1), 25.3 nm (d_2) and 24.6 nm (d_3), respectively. The diameters of Q1 and Q2 are estimated to be 26 nm (D_1) and 27.1 nm (D_2), respectively. The lengths of the GNRs are then estimated according to the darkfield scattering spectra shown in Fig. 1c. The lengths of G1, G2 and G3 are estimated to be 84.5 nm (l_1), 86 nm (l_2) and 86 nm (l_3), respectively. Finally, the gap widths between G1 and G3 and between G2 and G3 are estimated according to the measured excitation enhancement factors since the field enhancement effect in a gap is strongly related to the gap width. The gap width between G1 and G3 is estimated to be 29 nm (g_1), while the gap width between G2 and G3 is estimated to be 26 nm (g_2). These estimated structure parameters are used for the numerical simulations in the main text, except the structure with only Q1, where g_2 is estimated to be 30 nm by comparing the AFM image with that of the structure with both Q1 and Q2. Although not quantitatively accurate, simulations using these roughly estimated structure parameters can qualitatively reflect the features in the experiment. For these simulations, s_1 and s_2 are taken as 6 nm, as we find that the best excitation selectivity is obtained when s_1 and s_2 are around 6 nm.

The implementation of selective excitation and selective detection is quite robust to variations in structural parameters, although the optimal conditions (the excitation polarization for selective excitation and the polarizer angle for selective detection) and the corresponding optimal selectivity may change slightly. In Supplementary Table 1, we show some parameter modifications and their influences on selective excitations and emission polarizations. For the sake of analysis, the parameter modifications are based on a symmetric structure (denoted as #0 in the table) whose parameters are as follows (similar to the parameters estimated for the fabricated structure): $l_1 = l_2 = l_3 = 86$ nm; $d_1 = d_2 = d_3 = 25$ nm; $g_1 = g_2 = 25$ nm; $D_1 = D_2 = 25$ nm; $s_1 = s_2 = 6$ nm; $d_{x1} = d_{y1} = d_{z1} = d_{x2} = d_{y2} = d_{z2} = 0$ nm. Here d_{x1} (d_{x2}), d_{y1} (d_{y2}) and d_{z1} (d_{z2}) are the x-, y- and z-directional offset of Q1 (Q2) from the center of the whole silica sphere where the QD is encapsulated. From the table, we can see that with all these structure modifications, both the selective excitations and the selective detections can be achieved with sufficient selectivity. Both the degree of linear polarization (DOLP) and the polarization angle (ψ) of emission are insensitive to the parameter modifications, owing to that the emission wavelength (~808 nm) is off the plasmonic resonance. For all the parameter modifications, the DOLPs remain above 0.97 and the polarization angle difference between Q1 and Q2 is stable around 90°, facilitating selective detection with high transmittance simply using a linear polarizer. Parameter modification d_{y1} (see structures #12 and #13) influences the polarization angle of Q1 a bit more than other parameter modifications do. For all the parameter modifications, the excitation ratio keeps high enough to perform selective excitation, with the required excitation polarization (θ, φ) changing with parameter modifications. The modifications of s_1 (structures #8 and #9), d_{x1} (structures #10 and #11) and d_{z1} (structure #15) influence the excitation selectivity a bit more than the modification of other parameters, as they tend to degrade the dominance of the y-component for the local electric fields at the QDs (see the mechanism for selective excitation in Supplementary Note 2). A high excitation selectivity requires small x- and z-component electric field at the QDs. The modification of d_{z1} in the negative direction (structure #15) tends to degrade the excitation selectivity, whereas the modification in the positive direction (structure #14) slightly improves the excitation selectivity. This is attributed to the existence of the substrate. Without the

substrate, the z-component electric field is minimum at the center height of the structure and the field distribution is mirror-symmetric in the z-direction about the center height (see Supplementary Fig. 4a,b). When there is the substrate, the z-component electric field is minimum at a height several nanometers above the center height of the structure and the field distribution is no longer mirror-symmetric in the z-direction (see Supplementary Fig. 4c,d).

Supplementary Note 2 - Mechanism for selective excitation

An elliptically polarized excitation light can be viewed as a coherent combination of an x-polarized ($\leftrightarrow$) excitation light $E_0^{\leftrightarrow}\exp\left(\varphi_0^{\leftrightarrow}\right)\mathbf{n}_\mathrm{x}$ and a y-polarized ($\updownarrow$) excitation light $E_0^{\updownarrow}\exp\left(\varphi_0^{\updownarrow}\right)\mathbf{n}_\mathrm{y}$, where $E_0^{\leftrightarrow,\updownarrow}$ and $\varphi_0^{\leftrightarrow,\updownarrow}$ are the amplitude and phase of electric field and $\mathbf{n}_\mathrm{x,y}$ are the unit vectors. Therefore, the elliptical polarization can be fully described by parameters θ and φ, where θ is the arc tangent of the amplitude ratio $E_0^{\updownarrow}/E_0^{\leftrightarrow}$, φ is the phase difference $\varphi_0^{\updownarrow}-\varphi_0^{\leftrightarrow}$.

When illuminating with an elliptically polarized light, the source normalized local electric fields can be expressed as

$$\mathbf{E}=\cos\theta\cdot\mathbf{E}^{\leftrightarrow}+e^{\mathrm{i}\varphi}\sin\theta\cdot\mathbf{E}^{\updownarrow}\,, \tag{1}$$

where $\mathbf{E}^{\leftrightarrow}$ and $\mathbf{E}^{\updownarrow}$ are the source normalized local electric fields when illuminating with x-polarized ($\leftrightarrow$) and y-polarized ($\updownarrow$) light, respectively. Here, $\cos\theta\cdot\mathbf{E}^{\leftrightarrow}$ is the source-normalized local electric fields generated by the x-component of the elliptically polarized excitation light, and $e^{\mathrm{i}\varphi}\sin\theta\cdot\mathbf{E}^{\updownarrow}$ is the source-normalized local electric fields generated by the y-component of the elliptically polarized excitation light.

When illuminating with either x-polarized ($\leftrightarrow$) or y-polarized ($\updownarrow$) light, the source-normalized local field at both Q1 and Q2 ($\mathbf{E}^{\leftrightarrow,\updownarrow}(\mathrm{Q}i)$) has a strongly enhanced y-component $E_\mathrm{y}^{\leftrightarrow,\updownarrow}(\mathrm{Q}i)$ (Supplementary Fig. 5b,g) but very weak x-component $E_\mathrm{x}^{\leftrightarrow,\updownarrow}(\mathrm{Q}i)$ (Supplementary Fig. 5a,f) and z-component $E_\mathrm{z}^{\leftrightarrow,\updownarrow}(\mathrm{Q}i)$ (Supplementary Fig. 5c,h). That is, the y-component $E_\mathrm{y}^{\leftrightarrow,\updownarrow}(\mathrm{Q}i)$ is dominant in $\mathbf{E}^{\leftrightarrow,\updownarrow}(\mathrm{Q}i)$.

When illuminating with an elliptically polarized light, the y-component of the source-normalized electric field at Qi is

$$E_\mathrm{y}(\mathrm{Q}i)=\cos\theta\cdot E_\mathrm{y}^{\leftrightarrow}(\mathrm{Q}i)+e^{\mathrm{i}\varphi}\sin\theta\cdot E_\mathrm{y}^{\updownarrow}(\mathrm{Q}i)\,, \tag{2}$$

where $\cos\theta\cdot E_\mathrm{y}^{\leftrightarrow}(\mathrm{Q}i)$ is the source-normalized y-component electric field generated by the x-component of the elliptically polarized excitation light, and $e^{\mathrm{i}\varphi}\sin\theta\cdot E_\mathrm{y}^{\updownarrow}(\mathrm{Q}i)$ is the source-normalized y-component electric field generated by the y-component. If the elliptically polarized excitation light has polarization parameters

$$\theta=\arctan\left(\left|E_\mathrm{y}^{\leftrightarrow}(\mathrm{Q}i)\right|/\left|E_\mathrm{y}^{\updownarrow}(\mathrm{Q}i)\right|\right) \tag{3}$$

and

$$\varphi=\phi\left(E_\mathrm{y}^{\leftrightarrow}(\mathrm{Q}i)\right)-\phi\left(E_\mathrm{y}^{\updownarrow}(\mathrm{Q}i)\right)-180^\circ\,, \tag{4}$$

the generated y-component local electric fields $\cos\theta\cdot E_\mathrm{y}^{\leftrightarrow}(\mathrm{Q}i)$ and $e^{\mathrm{i}\varphi}\sin\theta\cdot E_\mathrm{y}^{\updownarrow}(\mathrm{Q}i)$ will have the same amplitude and are anti-phase. According to Supplementary Equation 2, we will have a complete destructive interference to produce a vanishing $E_\mathrm{y}(\mathrm{Q}i)$. As the y-component $E_\mathrm{y}^{\leftrightarrow,\updownarrow}(\mathrm{Q}i)$ is dominant in $\mathbf{E}^{\leftrightarrow,\updownarrow}(\mathrm{Q}i)$, the local electric field at Qi $\mathbf{E}(\mathrm{Q}i)$ is optimally suppressed this way. The amplitudes of the very weak x- and z- components determine how small $\mathbf{E}(\mathrm{Q}i)$ can be suppressed to.

We then consider the local field at the other QD Qj with the elliptically polarized excitation light defined by Supplementary Equations 3 and 4. The y-component of the source-normalized electric field at Qj is

$$E_{\mathrm{y}}(\mathrm{Q}j) = \cos\theta \cdot E_{\mathrm{y}}^{\leftrightarrow}(\mathrm{Q}j) + e^{\mathrm{i}\varphi}\sin\theta \cdot E_{\mathrm{y}}^{\updownarrow}(\mathrm{Q}j)\,. \tag{5}$$

Here $\cos\theta \cdot E_{\mathrm{y}}^{\leftrightarrow}(\mathrm{Q}j)$ is the source-normalized y-component electric field generated by the x-component of the elliptically polarized excitation light, and $e^{\mathrm{i}\varphi}\sin\theta \cdot E_{\mathrm{y}}^{\updownarrow}(\mathrm{Q}j)$ is the source-normalized y-component electric field generated by the y-component. Their amplitude ratio is

$$\frac{\left|e^{\mathrm{i}\varphi}\sin\theta \cdot E_{\mathrm{y}}^{\updownarrow}(\mathrm{Q}j)\right|}{\left|\cos\theta \cdot E_{\mathrm{y}}^{\leftrightarrow}(\mathrm{Q}j)\right|} = \tan\theta \cdot \frac{\left|E_{\mathrm{y}}^{\updownarrow}(\mathrm{Q}j)\right|}{\left|E_{\mathrm{y}}^{\leftrightarrow}(\mathrm{Q}j)\right|} = \frac{\left|E_{\mathrm{y}}^{\leftrightarrow}(\mathrm{Q}i)\right|}{\left|E_{\mathrm{y}}^{\leftrightarrow}(\mathrm{Q}j)\right|} \Bigg/ \frac{\left|E_{\mathrm{y}}^{\updownarrow}(\mathrm{Q}i)\right|}{\left|E_{\mathrm{y}}^{\updownarrow}(\mathrm{Q}j)\right|}\,, \tag{6}$$

which is close to 1, because the amplitude ratio between the local field at Q1 and at Q2 are similar for x- and y- polarized excitation. Their phase difference is

$$\begin{aligned}\phi\left(e^{\mathrm{i}\varphi}\sin\theta \cdot E_{\mathrm{y}}^{\updownarrow}(\mathrm{Q}j)\right) - \phi\left(\cos\theta \cdot E_{\mathrm{y}}^{\leftrightarrow}(\mathrm{Q}j)\right) &= \varphi + \phi\left(E_{\mathrm{y}}^{\updownarrow}(\mathrm{Q}j)\right) - \phi\left(E_{\mathrm{y}}^{\leftrightarrow}(\mathrm{Q}j)\right) \\ &= \phi\left(E_{\mathrm{y}}^{\leftrightarrow}(\mathrm{Q}i)\right) - \phi\left(E_{\mathrm{y}}^{\leftrightarrow}(\mathrm{Q}j)\right) + \phi\left(E_{\mathrm{y}}^{\updownarrow}(\mathrm{Q}j)\right) - \phi\left(E_{\mathrm{y}}^{\updownarrow}(\mathrm{Q}i)\right) - 180^{\circ}\end{aligned}\,, \tag{7}$$

which is close to 0, because $\phi\left(E_{\mathrm{y}}^{\leftrightarrow}(\mathrm{Q1})\right)$ and $\phi\left(E_{\mathrm{y}}^{\leftrightarrow}(\mathrm{Q2})\right)$ are roughly anti-phase (Supplementary Fig. 5d) while $\phi\left(E_{\mathrm{y}}^{\updownarrow}(\mathrm{Q1})\right)$ and $\phi\left(E_{\mathrm{y}}^{\updownarrow}(\mathrm{Q2})\right)$ are roughly in-phase (Supplementary Fig. 5i). Therefore, according to Supplementary Equation 5, the local field at Qj should experience constructive interference. Owing to the imperfect mirror-symmetry of the structure, $\phi\left(E_{\mathrm{y}}^{\leftrightarrow}(\mathrm{Q1})\right)$ and $\phi\left(E_{\mathrm{y}}^{\leftrightarrow}(\mathrm{Q2})\right)$ are not rigorously anti-phase and $\phi\left(E_{\mathrm{y}}^{\updownarrow}(\mathrm{Q1})\right)$ and $\phi\left(E_{\mathrm{y}}^{\updownarrow}(\mathrm{Q2})\right)$ are not rigorously in-phase. Therefore, the phase difference in Supplementary Equation 7 is not exactly 0 and the local field constructive interference at Qj is not optimal.

The combination of the destructive interference at one QD Qi and the constructive interference at the other QD Qj leads to selective excitation of Qj with high excitation selectivity. The condition (excitation polarization parameters θ and φ) for optimal selective excitation of Qj is just the condition for optimal suppression of the excitation of Qi, but not the condition for optimal excitation enhancement for Qj. With the condition for optimal selective excitation, the excitation enhancement for Qj may not be optimal. As the amplitudes of the very weak x- and z- components determine how small $\mathbf{E}(\mathrm{Q}i)$ can be suppressed to, they also determine the best excitation selectivity we can get.

Selective excitation of Q1 and Q2 can be realized in a broad range of wavelengths (by illuminating with wavelength-dependent elliptically polarized light) covering non-resonant and resonant excitations. Here we present selective excitation of Q2 (excitation suppression of Q1) in a broad range of wavelength. The source-normalized field amplitude spectra of the x- and z-component electric field at Q1 are shown in Supplementary Fig. 6a. The source-normalized field amplitude spectra of the y-component electric field at Q1 are shown in Supplementary Fig. 6b. In the broad range of wavelength, the y-component is dominant. The phase spectra of the y-component electric field at Q1 is shown in Supplementary Fig. 6c. Then the polarization parameters (θ, φ) for optimal excitation suppression of Q1 at different wavelengths can be obtained using the equations $\theta = \arctan\left(\left|E_{\mathrm{y}}^{\leftrightarrow}\right| / \left|E_{\mathrm{y}}^{\updownarrow}\right|\right)$ and $\varphi = \phi\left(E_{\mathrm{y}}^{\leftrightarrow}\right) - \phi\left(E_{\mathrm{y}}^{\updownarrow}\right) - 180^{\circ}$ as shown by the solid curves in Supplementary Fig. 6d. The excitation enhancement factors for Q1 and Q2 are shown in Supplementary Fig. 6e when excited with the optimal excitation polarizations at different wavelengths. The excitation of Q1 is well suppressed for a broad spectral range, while the

excitation of Q2 is strongly enhanced, which enables selective excitation with high selectivity for a broad spectral range as shown in Supplementary Fig. 6f. High selectivity exceeding 0.9995 (excitation ratio exceeding 4,000) can be achieved around 740 nm. At wavelengths shorter than 710 nm, the dominance of the y-component degrades (comparing between $\left|E_{\mathrm{x}}^{\leftrightarrow}\right|$ in Supplementary Fig. 6a and $\left|E_{\mathrm{y}}^{\leftrightarrow}\right|$ in Supplementary Fig. 6b), leading to relatively lower selectivity below 0.98 (excitation ratio below 100). In the wavelength range where the dominance of the y-component degrades, the optimal excitation condition determined using the equations $\theta = \arctan\left(\left|E_{\mathrm{y}}^{\leftrightarrow}\right| / \left|E_{\mathrm{y}}^{\updownarrow}\right|\right)$ and $\varphi = \phi\left(E_{\mathrm{y}}^{\leftrightarrow}\right) - \phi\left(E_{\mathrm{y}}^{\updownarrow}\right) - 180^{\circ}$ may deviate slightly from the actual optimal excitation condition (comparing between the solid curves and the dashed curves in Supplementary Fig. 6d-f, where the dashed curves are determined using the searching method described in Supplementary Note 5).

For understanding selective excitation in our design, two key points need to be addressed in more detail. The first key point is why the local fields at the QDs have very weak x- and z-components as compared with the dominant y-component (as shown in Supplementary Fig. 5a-c for x-polarized excitation and f-h for y-polarized excitation). If there is no substrate, the z-component at the QDs should be negligible because the structure is symmetric in the z-direction and the QDs are near the center height of the structure (i.e., the symmetry plane). Although the existence of the substrate breaks the symmetry, the QDs are still near the height with vanishing z-component electric field (cf. Supplementary Fig. 4 for the influence of the substrate on the z-component local fields). To explain the very weak x-components at the QDs, consider the electric field distribution around the end of a GNR in Supplementary Fig. 7a. We can see from the electric field vectors that the electric fields around the end cap of a GNR are roughly normal to the surface of the GNR. Therefore, at the QDs in the U-shaped gold nanostructure, either the electric field contributed by the y-oriented GNR (G1 for Q1 and G2 for Q2) or the electric field contributed by the x-oriented GNR (G3) should be roughly y-oriented with very weak x-component. Moreover, we view the U-shaped gold structure as two parts, one composed of G3 that is x-oriented and the other composed of G1 and G2 that are both y-oriented. And we simulate the electric field distributions separately for these two parts as shown in Supplementary Fig. 7. At the locations where the QDs should reside in the U-shaped nanosystem, the x-component electric fields are very weak for both structure parts and for both x- and y-polarized excitations (see Supplementary Fig. 7b,d,f,h).

The second key point is why the phase relation between the local fields at Q1 and Q2 is anti-phase when excited with x-polarized light while it is in-phase when excited with y-polarized light. When excited with x-polarized light, only the x-oriented GNR G3 is excited directly. The plasmonic oscillations in the y-oriented GNRs G1 and G2 are induced by the plasmonic oscillation in G3 through capacitive coupling with the right end and left end of G3, respectively. Therefore, the electric displacement vectors in G1 and G2 are in the opposite direction (as shown in Supplementary Fig. 5e). The electric field at Q1 is contributed by G1 and G3, while the electric field at Q2 is contributed by G2 and G3. Since the electric displacement vectors in G1 and G2 are in the opposite direction, the electric field contributed by G1 at Q1 and the electric field contributed by G2 at Q2 should be in the opposite direction. Moreover, the electric field contributed by G3 at Q1 and the electric field contributed by G3 at Q2 are also in the opposite direction (Supplementary Fig. 7a). Therefore, the total electric field at Q1 and Q2 are in the opposite direction and the phase of the y-component at Q1 and Q2 are anti-phase as shown in Supplementary Fig. 5d. When excited with y-polarized light, the y-oriented GNRs G1 and G2 are directly excited. Therefore, the electric displacement vectors in G1 and G2 are in the same direction (as shown in Supplementary Fig. 5j). Although G3 is capacitively coupled with G1 and G2 at its two ends

respectively, the induced current in G3 by G1 and that by G2 counteracts with each other and therefore the plasmonic oscillation in G3 is very weak as shown in Supplementary Fig. 5j. Therefore, the electric field at Q1 is contributed only by G1 and the electric field at Q2 is contributed only by G2. Since the electric displacement vectors in G1 and G2 are in the same direction, the electric field at Q1 and Q2 should be in the same direction and the phase of the y-component at Q1 and Q2 are in-phase as shown in Supplementary Fig. 5i.

Supplementary Note 3 - Purcell effect for QDs in the plasmonic nanostructure

The analysis of Purcell effect for a QD in a plasmonic nanostructure is a non-trivial problem, because the exciton in a QD is not a simple dipole, but has complicated fine structure levels[1]. Each fine structure level has its decay rate and transition orientation[2]. Therefore, the fluorescence decay or Purcell effect for a QD is influenced by its intrinsic fine structure and its orientation with respect to the plasmonic nanostructure. The Purcell effect determines the energy transfer from the excited QD to the plasmonic mode. The plasmonic mode further determines the polarization of photon radiation.

In the following, we assume that the QD is weakly excited so that the probability of excitation of biexcitons or multiexcitons can be neglected and we only need to consider the decay of monoexcitons. We also assume that thermalization is much faster than decay dynamics, even with Purcell effect, so that the decay dynamics can be described with an effective decay rate[3].

Influence of fine structure on Purcell effect

The intrinsic decay rate of a QD is the effective decay rate[3]

$$\gamma^0 = \sum_{i\in \mathrm{A}} \rho_i \gamma_i^0 + \sum_{j\in \mathrm{F}} \rho_j \gamma_j^0 , \tag{8}$$

where the first term is from the dipole-allowed modes, the second term is from the dipole-forbidden, phonon-assisted modes, ρ_i (ρ_j) is the population probability of the exciton state $i \in \mathrm{A}$ ($j \in \mathrm{F}$), γ_i^0 (γ_j^0) is the decay rate of the exciton state $i \in \mathrm{A}$ ($j \in \mathrm{F}$). Here $\sum_{i\in \mathrm{A}} \rho_i + \sum_{j\in \mathrm{F}} \rho_j = 100\%$. The decay probability from state $i \in \mathrm{A}$ ($j \in \mathrm{F}$) is

$$p_i^0 = \rho_i \gamma_i^0 / \gamma^0 \quad (p_j^0 = \rho_j \gamma_j^0 / \gamma^0). \tag{9}$$

Here $\sum_{i\in \mathrm{A}} p_i^0 + \sum_{j\in \mathrm{F}} p_j^0 = 100\%$.

At room temperature, there is always some dipole-allowed transition state considerably populated, therefore the emission from dipole-forbidden decays is negligible due to their extremely slow decay rate ($\sim \mu\mathrm{s}^{-1}$) as compared with that of dipole-allowed decays ($\sim \mathrm{ns}^{-1}$)[3]. Therefore, the effective decay rate can be simplified as

$$\gamma^0 = \sum_{i\in \mathrm{A}} \rho_i \gamma_i^0 . \tag{10}$$

When the QD is coupled to the plasmonic nanostructure, the decay rate becomes

$$\gamma = \sum_{i\in \mathrm{A}} \rho_i \gamma_i = \sum_{i\in \mathrm{A}} \rho_i f_i \gamma_i^0 , \tag{11}$$

where the decay rate γ_i is equal to $f_i\gamma_i^0$, due to the Purcell effect. The Purcell factors f_i are different for states with different transition wavelengths and different transition orientations, due to the wavelength dispersion and anisotropy of the partial local density of states in the plasmonic nanostructure. The effective Purcell factor that is experimentally measured can be expressed as

$$f=\frac{\gamma}{\gamma^0}=\frac{\sum_{i\in A}\rho_i f_i\gamma_i^0}{\gamma^0}=\sum_{i\in A}p_i^0 f_i\,, \tag{12}$$

where we can see that the effective Purcell factor is influenced by the intrinsic fine structure. The decay probability from state i is

$$p_i=\frac{\rho_i f_i\gamma_i^0}{\gamma}=\frac{p_i^0 f_i}{f}\,, \tag{13}$$

from which we can see that Purcell effect can influence the decay probabilities.

For spherical colloidal QDs, there are five dipole-allowed states: $\pm1\text{L}$, $\pm1\text{U}$ and 0U. The states $\pm1\text{L}$ and states $\pm1\text{U}$ are degenerate respectively. The states $+1\text{L}$ (-1L) and $+1\text{U}$ (-1U) emit σ^+ (σ^-) photons with right (left) circular polarization in the plane perpendicular to the c-axis of the nanocrystal, while the state 0U emits π photons with linear polarization along the c-axis (Supplementary Fig. 8a). The decay rate can be expressed as

$$\gamma^0=\rho_{1\text{L}}\gamma_{1\text{L}}^0+\rho_{1\text{U}}\gamma_{1\text{U}}^0+\rho_{0\text{U}}\gamma_{0\text{U}}^0\,, \tag{14}$$

where we have combined the degenerate states since $\rho_{+1\text{L(U)}}=\rho_{-1\text{L(U)}}=\frac{1}{2}\rho_{1\text{L(U)}}$ and $\gamma_{+1\text{L(U)}}^0=\gamma_{-1\text{L(U)}}^0=\gamma_{1\text{L(U)}}^0$. The combination of the transitions from $\pm1\text{L}$ (or $\pm1\text{U}$) correspond to a 2D-dipole in the plane perpendicular to the c-axis of the nanocrystal (Supplementary Fig. 8a)[2,4]. The decay probability from these 2D-dipole transitions is

$$p_{2\text{D}}^0=\frac{\left(\rho_{1\text{L}}\gamma_{1\text{L}}^0+\rho_{1\text{U}}\gamma_{1\text{U}}^0\right)}{\left(\rho_{1\text{L}}\gamma_{1\text{L}}^0+\rho_{1\text{U}}\gamma_{1\text{U}}^0\right)+\rho_{0\text{U}}\gamma_{0\text{U}}^0}\,, \tag{15}$$

while the decay probability from the linear dipole transition along the c-axis is

$$p_{0\text{U}}^0=\frac{\rho_{0\text{U}}\gamma_{0\text{U}}^0}{\left(\rho_{1\text{L}}\gamma_{1\text{L}}^0+\rho_{1\text{U}}\gamma_{1\text{U}}^0\right)+\rho_{0\text{U}}\gamma_{0\text{U}}^0}\,. \tag{16}$$

When QD is coupled to a plasmonic nanostructure, the decay rate of the QD becomes

$$\gamma=\rho_{1\text{L}}f_{1\text{L}}\gamma_{1\text{L}}^0+\rho_{1\text{U}}f_{1\text{U}}\gamma_{1\text{U}}^0+\rho_{0\text{U}}f_{0\text{U}}\gamma_{0\text{U}}^0\,, \tag{17}$$

where $f_{1\text{L}}$, $f_{1\text{U}}$ and $f_{0\text{U}}$ are the Purcell factors for corresponding transitions. If we neglect the wavelength dispersion of the Purcell effect among the fine structure levels, we have $f_{1\text{L}}=f_{1\text{U}}=f_{2\text{D}}$. Then Supplementary Equation 17 can be expressed as

$$\gamma=f_{2\text{D}}\left(\rho_{1\text{L}}\gamma_{1\text{L}}^0+\rho_{1\text{U}}\gamma_{1\text{U}}^0\right)+f_{0\text{U}}\left(\rho_{0\text{U}}\gamma_{0\text{U}}^0\right). \tag{18}$$

Then the effective Purcell factor that is experimentally measured can be expressed as

$$f=\frac{\gamma}{\gamma^0}=\frac{f_{2\text{D}}\left(\rho_{1\text{L}}\gamma_{1\text{L}}^0+\rho_{1\text{U}}\gamma_{1\text{U}}^0\right)+f_{0\text{U}}\left(\rho_{0\text{U}}\gamma_{0\text{U}}^0\right)}{\left(\rho_{1\text{L}}\gamma_{1\text{L}}^0+\rho_{1\text{U}}\gamma_{1\text{U}}^0\right)+\rho_{0\text{U}}\gamma_{0\text{U}}^0}=p_{2\text{D}}^0 f_{2\text{D}}+p_{0\text{U}}^0 f_{0\text{U}}\,, \tag{19}$$

from which we can see that the effective Purcell effect is influenced by the intrinsic decay probabilities from the 2D transition dipole and the c-axis transition dipole. For spherical QDs $p_{0\text{U}}^0$ is much smaller

than p_{2D}^0, hence the c-axis is also called 'dark axis'[2]. With the Purcell effect, the decay probability from these 2D-dipole transitions is modified to

$$p_{2D} = \frac{f_{2D}\left(\rho_{1L}\gamma_{1L}^0 + \rho_{1U}\gamma_{1U}^0\right)}{f_{2D}\left(\rho_{1L}\gamma_{1L}^0 + \rho_{1U}\gamma_{1U}^0\right) + f_{0U}\left(\rho_{0U}\gamma_{0U}^0\right)} = \frac{p_{2D}^0 f_{2D}}{f}, \tag{20}$$

while the decay probability from the linear dipole transition along the c-axis is modified to

$$p_{0U} = \frac{f_{0U}\left(\rho_{0U}\gamma_{0U}^0\right)}{f_{2D}\left(\rho_{1L}\gamma_{1L}^0 + \rho_{1U}\gamma_{1U}^0\right) + f_{0U}\left(\rho_{0U}\gamma_{0U}^0\right)} = \frac{p_{0U}^0 f_{0U}}{f}. \tag{21}$$

From Supplementary Equations 20 and 21, we can see that the 'dark axis' may be made bright by the Purcell effect, as long as the Purcell factor f_{0U} is much larger than f_{2D}.

Influence of orientation on Purcell effect

The Purcell factors f_{2D} and f_{0U} are influenced by the orientation of the QD in the plasmonic nanostructure, and can be determined as:

$$f_{2D} = \frac{p_{x,2D}^0}{p_{2D}^0} f_x + \frac{p_{y,2D}^0}{p_{2D}^0} f_y + \frac{p_{z,2D}^0}{p_{2D}^0} f_z, \tag{22}$$

$$f_{0U} = \frac{p_{x,0U}^0}{p_{0U}^0} f_x + \frac{p_{y,0U}^0}{p_{0U}^0} f_y + \frac{p_{z,0U}^0}{p_{0U}^0} f_z. \tag{23}$$

Here f_x, f_y and f_z are the Purcell factors for x-, y-, and z-oriented transition dipoles. $p_{x,2D}^0$, $p_{y,2D}^0$ and $p_{z,2D}^0$ are the intrinsic decay probabilities projected to x-, y- and z-axis for the 2D transition dipole, while $p_{x,0U}^0$, $p_{y,0U}^0$ and $p_{z,0U}^0$ are the intrinsic decay probabilities projected to x-, y- and z-axis for the linear transition dipole along the c-axis. Substituting Supplementary Equations 22 and 23 into Supplementary Equation 19, we can obtain the effective Purcell factor f using the Purcell factors f_x, f_y, f_z and the intrinsic decay probabilities projected to x-, y- and z-axis:

$$f = p_x^0 f_x + p_y^0 f_y + p_z^0 f_z, \tag{24}$$

where $p_x^0 = p_{x,2D}^0 + p_{x,0U}^0$, $p_y^0 = p_{y,2D}^0 + p_{y,0U}^0$, $p_z^0 = p_{z,2D}^0 + p_{z,0U}^0$.

For a QD oriented as shown in Supplementary Fig. 8a, the intrinsic decay probabilities projected to x-, y- and z-axis are

$$\begin{aligned}
p_{x,2D}^0 &= \tfrac{1}{2} p_{2D}^0 \left[(\cos\beta\cos\alpha)^2 + (\sin\alpha)^2\right], \\
p_{y,2D}^0 &= \tfrac{1}{2} p_{2D}^0 \left[(\cos\beta\sin\alpha)^2 + (\cos\alpha)^2\right], \\
p_{z,2D}^0 &= \tfrac{1}{2} p_{2D}^0 (\sin\beta)^2, \\
p_{x,0U}^0 &= p_{0U}^0 (\sin\beta\cos\alpha)^2, \\
p_{y,0U}^0 &= p_{0U}^0 (\sin\beta\sin\alpha)^2, \\
p_{z,0U}^0 &= p_{0U}^0 (\cos\beta)^2.
\end{aligned} \tag{25}$$

The Purcell factors f_x, f_y and f_z at Q1 and Q2 are numerically calculated and plotted in Supplementary Fig. 8b,c. With the Purcell effects, the decay probabilities projected to x-, y- and z-axis are modified to

$$p_x = \frac{p_x^0 f_x}{f}, \quad p_y = \frac{p_y^0 f_y}{f}, \quad p_z = \frac{p_z^0 f_z}{f}. \tag{26}$$

Simulated and measured Purcell effects

Since p_{0U}^0 is much smaller than p_{2D}^0, the effective Purcell factor f is maximal when the 'dark axis' is in the xz plane and is minimal when the 'dark axis' is along the y axis. From simulation, we can see that f_x and f_z are much smaller than f_y, thus the maximal effective Purcell factor can be approximately expressed as $f_{max} = \frac{1}{2} p_{2D}^0 f_y$. Then we can get the upper limit of the effective Purcell factor as $\frac{1}{2} f_y$, which is ~93 for Q1 and ~145 for Q2 (see f_y at 808nm in Supplementary Fig. 8b). The experimentally measured effective Purcell factor is ~45±3 for Q1, which is significantly below the upper limit implying a non-optimal orientation. The experimentally measured effective Purcell factor is ~132±8 for Q2, which approaches the upper limit implying a near-optimal orientation.

As shown in Supplementary Fig. 9a, the lifetime of Q1 is shorter when both Q1 and Q2 are in the nanosystem (corresponding to the structure measured in Fig. 3 and 4) than when only Q1 is in the nanosystem (corresponding to the structure measured in Fig. 2 of the main text). We attribute this to the change of structure parameters during the process of moving Q2 into the nanosystem. As shown by the simulated Purcell factors in Supplementary Fig. 9b, both the refractive index of the silica-encapsulated Q2 (simply modelled here as a silica sphere) and the decrease of g_2 (gap width between G2 and G3) can cause a red shift of the plasmonic resonance and consequently increase the Purcell factor at the emission wavelength ~808 nm. There is also possibility that the gap width between G1 and G3 is slightly altered since pushing Q2 or G2 during the manipulation process may also move G3 through direct or indirect contact. We stress here that the influence from the existence of Q2 is due to its refractive index, but not the energy transfer. As we will analyze in Supplementary Note 4, the energy transfer rate should be much smaller than the spontaneous emission rate and therefore the existence of energy transfer should not influence the lifetime measurement.

Since the lifetime curves are nearly mono-exponential under selective excitation or selective detection and the minor decay component can be attributed to the other QD due to the finite selectivity (see Supplementary Note 6 for the exponential fitting of the lifetime curves), the decay of each QD can be regarded as mono-exponential. When only Q1 is in the nanosystem, the measured lifetime curve is indeed mono-exponential (Supplementary Fig. 9a). With strong Purcell effects, the decay dynamics of the QDs remains mono-exponential. The mono-exponential decay behaviours are expected in our measurement, for two reasons. First, the QDs are weakly excited, so the probability of excitation of biexcitons or multiexcitons can be neglected and the measured decay dynamics is of monoexcitons. Second, at room temperature the decay dynamics is still much slower than thermalization, so the decay dynamics can be well described with an effective decay rate[3], which is consistence with our analysis above (Supplementary Equation 11)

Emission polarization

Since very large effective Purcell factors are experimentally measured while f_x and f_z are very small, we can conclude from Supplementary Equation 24 that $p_y^0 f_y / f$ is near 100%. Then we can further conclude from Supplementary Equation 26 that p_y is near 100%, which means that nearly all the energy from the exciton decay is extracted to the plasmonic mode excited by a y-oriented dipole as shown in Supplementary Fig. 8d (the same as Fig. 1g) for Q2 and Supplementary Fig. 8e (the same as Fig. 1f) for Q1. This plasmonic mode further determines the far-field emission polarization. Therefore,

as long as the Purcell factor for Q1 (Q2) is much larger than 1, the orientation of Q1 (Q2) will not influence its emission polarization. Its emission polarization should be the same as that of a y-oriented 1D dipole emitter at its location. To numerically calculate the far-field polarization, we use a y-oriented 1D dipole at the location of the QD and simulate the near field distribution in a plane slightly below the plasmonic nanostructure and perform a far-field projection routine. The calculated far-field polarizations are in good agreement with the experimental results, as shown in Fig. 2b and Fig. 3b.

It is the distinct locations in the nanosystem that makes Q1 and Q2 couple to distinct plasmonic modes and consequently radiate with entirely different polarization states (roughly orthogonal to each other). To confirm this, we re-assemble the structure by moving the GNRs (Q1 is not moved) so that Q1 locates at the gap between G2 and G3 (which is Q2's location before the re-assembly) as shown by the AFM image in the lower inset in Supplementary Fig. 10 (the tiny bump on G3 is a fragment that sticks during the manipulation). When Q1 is at the gap between G1 and G3 (i.e., before the re-assembly), the polarization angle is ~46° (blue experimental data points and simulated continuous curve in Supplementary Fig. 10 or Fig. 2b). When Q1 is at the gap between G2 and G3 (i.e., after the re-assembly), the polarization angle changes to ~132° (red experimental data points and simulated continuous curve in Supplementary Fig. 10), which is very near the polarization angle of Q2 shown in Fig. 3b (for comparison, also shown with green 'x'-shaped data points in Supplementary Fig. 10).

The GNR farthest from the QD affects the far-field polarization. Before G2 is added, the far-field emission polarization of Q1 is linearly polarized with a polarization angle of ~52° (red experimental data points and simulated continuous curve in Supplementary Fig. 11a), while after G2 is added, the polarization angle rotates to ~46° (blue experimental data points and simulated continuous curve in Supplementary Fig. 11a). The electric field coupled to G2 (comparing field profile in panel c with that in panel b in Supplementary Fig. 11) is roughly anti-phase with the electric field in G1 and thus reduces the y-polarized component in the far-field radiation, which explains the slight rotation of polarization angle.

Supplementary Note 4 - Energy transfer between the QDs

For simplicity, here we model the emitters as point dipoles, one as the donor and the other as the acceptor. Further more, the dipoles are assumed to have the optimal orientations for energy transfer. For dipole-dipole energy transfer at subwavelength distance, the optimal orientation is pointing from donor to acceptor, as shown in the lower two insets in Supplementary Fig. 12a. For energy transfer with the plasmonic nanostructure, the optimal orientation is the y-direction, as shown in the upper inset in Supplementary Fig. 12a. Energy transfer rate γ_{ET} scale with $\left|\mathbf{n}_{\mathrm{A}}\cdot\vec{\mathbf{G}}(\mathbf{r}_{\mathrm{D}},\mathbf{r}_{\mathrm{A}})\mathbf{n}_{\mathrm{D}}\right|^2$, that is,

$$\gamma_{\mathrm{ET}}\propto\left|\mathbf{n}_{\mathrm{A}}\cdot\vec{\mathbf{G}}(\mathbf{r}_{\mathrm{D}},\mathbf{r}_{\mathrm{A}})\mathbf{n}_{\mathrm{D}}\right|^2, \tag{27}$$

where $\mathbf{n}_{\mathrm{D}}$ ($\mathbf{n}_{\mathrm{A}}$) is the unit vector denoting the dipole orientation of the donor (acceptor), $\mathbf{r}_{\mathrm{D}}$ ($\mathbf{r}_{\mathrm{A}}$) is the position of the donor (acceptor) and $\vec{\mathbf{G}}(\mathbf{r}_{\mathrm{D}},\mathbf{r}_{\mathrm{A}})$ is the Green's function[5]. In Supplementary Fig. 12a, we show the numerically simulated value of $\left|\mathbf{n}_{\mathrm{A}}\cdot\vec{\mathbf{G}}(\mathbf{r}_{\mathrm{D}},\mathbf{r}_{\mathrm{A}})\mathbf{n}_{\mathrm{D}}\right|^2$ (as a function of the wavelength of energy transfer) for three different cases. We can see that with the gold nanostructure, the value of $\left|\mathbf{n}_{\mathrm{A}}\cdot\vec{\mathbf{G}}(\mathbf{r}_{\mathrm{D}},\mathbf{r}_{\mathrm{A}})\mathbf{n}_{\mathrm{D}}\right|^2$ is strongly enhanced (red solid curve) as compared with the case without the gold nanostructure (blue dashed curve). The energy transfer rate enhancement factor f_{ET} is shown in

Supplementary Fig. 12b. At the wavelength of ~808 nm (i.e., the emission wavelength of the QDs used in our experiment), the enhancement factor is ~540. With this enhancement, the energy transfer rate between the two dipole emitters (~61 nm apart) coupled with the gold nanostructure will be the same as that between two dipole emitters ~22 nm apart (without the gold nanostructure), as shown by the intersection of the red solid curve and the blue solid curve at ~808 nm in Supplementary Fig. 12a.

In the experiment, the situation is more complex. Most importantly, the QDs has to be modelled as a combination of a 2D dipole and a 1D dipole, and the orientations of the QDs may deviate from the ideal case, which will significantly reduce both the Purcell effect and the enhancement factor of the energy transfer rate. For simplicity, the reduction can be effectively attributed to the orientation deviation of a 1D dipole from the y direction. Suppose that the donor (acceptor) dipole emitter μ_D (μ_A) deviate from the y direction by an angle of θ_D (θ_A). Recall that the Purcell factor

$$f_{\text{Purcell}} \propto \mathbf{n}_\mu \cdot \text{Im}\left\{\vec{\vec{\mathbf{G}}}(\mathbf{r}_0,\mathbf{r}_0)\right\} \cdot \mathbf{n}_\mu , \tag{28}$$

while the enhancement factor for the energy transfer rate

$$f_{\text{ET}} \propto \left| \mathbf{n}_A \cdot \vec{\vec{\mathbf{G}}}(\mathbf{r}_D,\mathbf{r}_A)\mathbf{n}_D \right|^2 . \tag{29}$$

In the nanosystem, for both $\vec{\vec{\mathbf{G}}}(\mathbf{r}_0,\mathbf{r}_0)$ and $\vec{\vec{\mathbf{G}}}(\mathbf{r}_D,\mathbf{r}_A)$, other components are negligible as compared with the component G_{yy}. Therefore, the Purcell factor for the donor (acceptor) scales with $\cos^2\theta_D$ ($\cos^2\theta_A$) while the enhancement factor of the energy transfer rate scales with $\cos^2\theta_D \cdot \cos^2\theta_A$, that is,

$$f_D \propto \cos^2\theta_D , \tag{30}$$

$$f_A \propto \cos^2\theta_A , \tag{31}$$

$$f_{\text{ET}} \propto \cos^2\theta_D \cdot \cos^2\theta_A . \tag{32}$$

To roughly estimate the enhancement of energy transfer rate, we compare theoretical Purcell factors with the experimentally observed Purcell factors. The theoretical Purcell factor (for the emission wavelength of ~808 nm) is ~188 for the dipole emitter μ_1 and ~290 for the dipole emitter μ_2 (Supplementary Fig. 12c), while in the experiment we observe a Purcell factor of ~45$\pm$3 for Q1 and a Purcell factor of ~132$\pm$8 for Q2, which are reduced with a factor of ~4.2 and ~2.2 respectively. Then we can roughly estimate that the enhancement factor of the energy transfer rate is reduced from the theoretical value (~540) by a factor of ~9.2 (the product of the reduction factors ~4.2 and ~2.2) to ~59.

Although the energy transfer rate is expected to be strongly enhanced in the nanosystem, it is still much smaller than the enhanced spontaneous emission rates and therefore, considering the competition between the energy transfer and the spontaneous emission of the donor[6], the energy transfer efficiency is so low that we can safely neglect the energy transfer in our experiment. For dipole-dipole energy transfer in homogenous free-space, the energy transfer rate γ_{ET} decays rapidly with donor-acceptor distance R and can be expressed as

$$\gamma_{\text{ET}} = \gamma_0 \cdot \left(\frac{R_0}{R}\right)^6 , \tag{33}$$

where γ_0 is the intrinsic decay rate of the donor (in the absence of the acceptor) and R_0 is the Förster radius. R_0 is typically in the range of 2-9 nm[7]. Since the energy transfer rate is enhanced to be the same as that between two dipole emitters ~22 nm apart, we can estimate the energy transfer rate to be

$$\gamma_{\mathrm{ET}} \le \gamma_0 \cdot \left(\frac{9\ \mathrm{nm}}{22\ \mathrm{nm}}\right)^6 = \frac{\gamma_0}{213} . \tag{34}$$

At the same time, the spontaneous emission rates of both dipole emitters are also strongly enhanced as shown in Supplementary Fig. 12c. The spontaneous emission rate of dipole emitter μ_1 is enhanced to be $\gamma_1 = 188\ \gamma_0$ and the emission rate of the dipole emitter μ_2 is enhanced to be $\gamma_2 = 290\ \gamma_0$. So we have

$$\gamma_{\mathrm{ET}} < \frac{\gamma_i}{4\times10^4}\ (i = 1,2) . \tag{35}$$

Therefore, the energy transfer efficiency E_i (i denotes that μ_i is the donor), defined as the fraction of energy transferred to the acceptor compared to the total energy released from the decay of the donor

$$E_i = \frac{\gamma_{\mathrm{ET}}}{\gamma_{\mathrm{ET}} + \gamma_i} , \tag{36}$$

is estimated to be lower than 2.5×10^{-5}. As the Purcell factor for the donor (acceptor) scales with $\cos^2\theta_{\mathrm{D}}$ ($\cos^2\theta_{\mathrm{A}}$) and the enhancement factor of the energy transfer rate scales with $\cos^2\theta_{\mathrm{D}} \cdot \cos^2\theta_{\mathrm{A}}$ (Supplementary Equations 30-32), if we consider the orientation deviation angles of the donor and acceptor dipole emitter from the optimal y-orientation θ_{D} and θ_{A}, the energy transfer efficiency would be even lower. Such a low energy transfer efficiency indicates that the energy transfer can be neglected in the decay of the emitters and will not influence the characterization of the emission properties of the emitters. On the other hand, although we can selectively excite the donor and selectively detect the acceptor, either the excitation selectivity or the detection selectivity in the experiment is not high enough to enable the characterization of the energy transfer. With the finite excitation selectivity of ~0.96, although the direct excitation of the acceptor is only ~1/50 of that of the selectively excited donor, the energy transfer from the donor to the acceptor would still be much weaker than the direct excitation of the acceptor. With the finite detection selectivity of ~0.96, although most of the emission from the donor is blocked, the detected emission from the energy transfer would still be much less than the directly detected emission from the donor.

From Supplementary Equations 28 and 29, we see that the energy transfer enhancement factor scales quadratically with the Green function while the Purcell factor scales linearly with the Green function (the imaginary part). If the Green functions are further enhanced by shrinking the gap size of the plasmonic structure, the energy transfer enhancement factor can be made much larger than the Purcell factor, and then the energy transfer efficiency (Supplementary Equation 36) can be enhanced.

Supplementary Note 5 - Finding optimal polarization for selective excitation and optimal polarizer angle for selective detection

To perform far-field selective excitation and far-field selective detection, the key task is to experimentally find, for each QD, the optimal polarization for excitation suppression and optimal polarizer angle for emission blocking.

When only one QD is in the system, we find the optimal excitation polarization for excitation suppression by successively searching the elliptical polarization parameters φ and θ to minimize the emission intensity. First, we set θ to a moderate value θ_0 (e.g., 45°) and scan φ to find the optimal

φ that minimizes the emission intensity. Then, we set φ to this optimal φ and scan θ to find the optimal θ that minimizes the emission intensity.

As long as the y-component local fields $E_y^{\leftrightarrow}$ and $E_y^{\updownarrow}$ are dominant at the QDs, this searching method is theoretically equivalent to the direct calculation method using $\theta = \arctan\left(\left|E_y^{\leftrightarrow}\right| / \left|E_y^{\updownarrow}\right|\right)$ and $\varphi = \phi\left(E_y^{\leftrightarrow}\right) - \phi\left(E_y^{\updownarrow}\right) - 180^\circ$ (described in Supplementary Note 2). Numerical simulations show that this searching method gives results (dashed curves in Supplementary Fig. 6d-f) in agreement with those given by direct calculation (solid curves in Supplementary Fig. 6d-f), and slightly better results at wavelengths shorter than 710 nm where the dominance of the y-component degrades.

When both QDs are in the system, if we use the emission intensity as the figure of merit for optimization of the excitation polarization, there is mutual dependence between determination of the optimal polarization for selective excitation and determination of the optimal polarizer angle for selective detection. To find the optimal excitation polarization to suppress either QD, we have to selectively detect the QD to minimize its emission intensity. To find the optimal polarizer angle to selectively detect either QD, we have to selectively excite the QD to measure its emission polarization.

This mutual dependence can be avoided if the objective parameter for optimization of the excitation polarization does not rely on selective detection. The degree of polarization (DOP) is such a feasible objective parameter, which is very sensitive to incoherent mixing of photons from Q1 and Q2 (since the emissions from Q1 and Q2 are designed to have pure polarizations well separated on the Poincaré sphere) and can be conveniently obtained by measuring the Stokes parameters.

In this work, we use the emission intensity as the objective parameter for optimization of the excitation polarization. We overcome the mutual dependence by iteratively optimizing the polarization for selective excitation and the polarizer angle for selective detection until stable values emerge after a small number of iterations, as illustrated in Supplementary Fig. 13, where panel a illustrates the optimization process for selective excitation of Q1 (i.e., excitation suppression of Q2) and selective detection of Q2 (i.e., blocking the emission of Q1), and panel b illustrates the optimization process for selective excitation of Q2 (i.e., excitation suppression of Q1) and selective detection of Q1 (i.e., blocking the emission of Q2). The superscripts in brackets (i) denotes the current round number of iteration. Since the processes in panel a and b are similar, here we describe in detail only the process in panel a. Before we start the optimization, we have to first decide $\psi_{Q1}^{(1)}$ (for the first round of iteration, the superscripts i is 1) as the initial value of the emission polarization angle of Q1 to start the optimization. Emission polarization angle from preliminary numerical simulation is a good candidate for $\psi_{Q1}^{(1)}$. Later, we will see that the optimization process permits a quite wide range of the initial value $\psi_{Q1}^{(1)}$. From the emission polarization angle of Q1 $\psi_{Q1}^{(i)}$ (decided as a initial value for $i = 1$ or produced from the previous iteration for $i > 1$), the polarizer angle α is set accordingly to $\psi_{Q1}^{(i)} + 90^\circ$. This way, the transmittance for Q2 $T_{Q2}\left(\psi_{Q1}^{(i)} + 90^\circ\right)$ is larger than the transmittance for Q1 $T_{Q1}\left(\psi_{Q1}^{(i)} + 90^\circ\right)$, so that Q2 is selectively detected with a certain selectivity. Here $T_{Q1}(\alpha)$ and $T_{Q2}(\alpha)$ are functions of the polarizer angle α, and are related to the actual emission polarization parameters of Q1 (ψ_{Q1}, χ_{Q1}) and Q2 (ψ_{Q2}, χ_{Q2}), respectively:

$$T_{Q1}(\alpha) = \frac{1}{1+\tan^2 \chi_{Q1}} \cos^2\left(\alpha - \psi_{Q1}\right) + \frac{\tan^2 \chi_{Q1}}{1+\tan^2 \chi_{Q1}} \sin^2\left(\alpha - \psi_{Q1}\right), \quad (37)$$

$$T_{Q2}(\alpha) = \frac{1}{1+\tan^2\chi_{Q2}}\cos^2(\alpha-\psi_{Q2}) + \frac{\tan^2\chi_{Q2}}{1+\tan^2\chi_{Q2}}\sin^2(\alpha-\psi_{Q2}) . \tag{38}$$

Under this detection condition, we can then find the optimal excitation polarization by successively searching the elliptical polarization parameters φ and θ that minimize the detected emission intensity. First, set θ to a moderate value θ_0 (e.g., 45°) and scan φ to find the optimal φ that minimizes the detected emission intensity. Due to finite detection selectivity, the detected emission intensity includes contributions from both Q1 and Q2 and can be expressed as (normalized)

$$g_1^{(i)}(\varphi) = f_{Q1}(\theta_0,\varphi)\cdot T_{Q1}\left(\psi_{Q1}^{(i)}+90^\circ\right) + f_{Q2}(\theta_0,\varphi)\cdot T_{Q2}\left(\psi_{Q1}^{(i)}+90^\circ\right) , \tag{39}$$

where $f_{Q1}(\theta,\varphi)$ and $f_{Q2}(\theta,\varphi)$ are the excitation enhancement factors for Q1 and Q2 as functions of the excitation polarization parameters (θ,φ):

$$\begin{aligned} f_{Q1}(\theta,\varphi) = &\left[\cos\theta\cdot E_x^{\leftrightarrow}(Q1) + e^{i\varphi}\sin\theta\cdot E_x^{\updownarrow}(Q1)\right]^2 \\ &+\left[\cos\theta\cdot E_y^{\leftrightarrow}(Q1) + e^{i\varphi}\sin\theta\cdot E_y^{\updownarrow}(Q1)\right]^2 \ , \\ &+\left[\cos\theta\cdot E_z^{\leftrightarrow}(Q1) + e^{i\varphi}\sin\theta\cdot E_z^{\updownarrow}(Q1)\right]^2 \end{aligned} \tag{40}$$

$$\begin{aligned} f_{Q2}(\theta,\varphi) = &\left[\cos\theta\cdot E_x^{\leftrightarrow}(Q2) + e^{i\varphi}\sin\theta\cdot E_x^{\updownarrow}(Q2)\right]^2 \\ &+\left[\cos\theta\cdot E_y^{\leftrightarrow}(Q2) + e^{i\varphi}\sin\theta\cdot E_y^{\updownarrow}(Q2)\right]^2 \ . \\ &+\left[\cos\theta\cdot E_z^{\leftrightarrow}(Q2) + e^{i\varphi}\sin\theta\cdot E_z^{\updownarrow}(Q2)\right]^2 \end{aligned} \tag{41}$$

The found optimal φ is denoted as $\varphi_1^{(i)}$. Then set φ to this optimal value $\varphi_1^{(i)}$ and scan θ to find the optimal θ that minimizes the detected emission intensity (normalized)

$$h_1^{(i)}(\theta) = f_{Q1}\left(\theta,\varphi_1^{(i)}\right)\cdot T_{Q1}\left(\psi_{Q1}^{(i)}+90^\circ\right) + f_{Q2}\left(\theta,\varphi_1^{(i)}\right)\cdot T_{Q2}\left(\psi_{Q1}^{(i)}+90^\circ\right) . \tag{42}$$

The found optimal θ is denoted as $\theta_1^{(i)}$. This way, with the excitation polarization parameters ($\theta_1^{(i)},\varphi_1^{(i)}$), the excitation enhancement factors for Q1 $f_{Q1}\left(\theta_1^{(i)},\varphi_1^{(i)}\right)$ is larger than the excitation enhancement factors for Q2 $f_{Q2}\left(\theta_1^{(i)},\varphi_1^{(i)}\right)$, that is, Q2 is suppressed to a certain degree and Q1 is selectively excited with a certain selectivity. Under this excitation condition, we then scan the polarizer angle α to find the polarizer angle that minimizes the detected emission. Due to finite excitation selectivity, the detected emission includes contributions from both Q1 and Q2 and can be expressed as (normalized)

$$v_1^{(i)}(\alpha) = f_{Q1}\left(\theta_1^{(i)},\varphi_1^{(i)}\right)\cdot T_{Q1}(\alpha) + f_{Q2}\left(\theta_1^{(i)},\varphi_1^{(i)}\right)\cdot T_{Q2}(\alpha) . \tag{43}$$

From the found polarizer angle α, we can get the polarization angle $\psi_{Q1}^{(i+1)} = \alpha - 90^\circ$. From $\psi_{Q1}^{(i+1)}$ we can then start another iteration described above until convergence is achieved for both $\psi_{Q1}^{(i)}$ and ($\theta_1^{(i)},\varphi_1^{(i)}$). The convergence speed is fast enough for our structure that the finally optimized excitation polarization and polarizer angle can be obtained within a small number of iterations if the starting trial value of the polarizer angle ($\psi_{Q1}^{(1)}+90^\circ$) doesn't deviate too much from the optimal value ($\psi_{Q1}+90^\circ$).

We theoretically demonstrate in Supplementary Fig. 14 the optimization process for the structure simulated in the main text. In Supplementary Fig. 14a, we start from a $\psi_{Q1}^{(1)}$ that deviates $20°$ from the

actual polarization angle ψ_{Q1}. This $\psi_{Q1}^{(1)}$ results in a transmittance ratio $T_{Q2}\left(\psi_{Q1}^{(1)}+90^\circ\right)/T_{Q1}\left(\psi_{Q1}^{(1)}+90^\circ\right)$ of 7.5. With such a moderate transmittance ratio, we find excitation polarization parameters ($\theta_1^{(1)},\varphi_1^{(1)}$) that deviate only slightly from the actual optimal excitation parameters (θ_1,φ_1) and can already be used as the final optimal parameters. Then with the excitation polarization parameters, high excitation selectivity is achieved and we can subsequently find $\psi_{Q1}^{(2)}$ with negligible deviation from the actual polarization angle ψ_{Q1}. Using $\psi_{Q1}^{(2)}$, the excitation polarization parameters ($\theta_1^{(2)},\varphi_1^{(2)}$) can further be found with negligible deviation from (θ_1,φ_1). In Supplementary Fig. 14b, we start from another $\psi_{Q1}^{(1)}$ that deviates an even larger angle (40 °) from ψ_{Q1}. The transmittance ratio $T_{Q2}\left(\psi_{Q1}^{(1)}+90^\circ\right)/T_{Q1}\left(\psi_{Q1}^{(1)}+90^\circ\right)$ is only 1.64. Nevertheless, with such a small transmittance ratio, we can still get excitation polarization parameters ($\theta_1^{(1)},\varphi_1^{(1)}$) that deviate not too much from the optimal excitation parameters (θ_1,φ_1). The parameters ($\theta_1^{(1)},\varphi_1^{(1)}$) can already offer efficient excitation selectivity to subsequently get a $\psi_{Q1}^{(2)}$ with negligible deviation from the actual polarization angle ψ_{Q1}. Comparing between Supplementary Fig. 14a and b, we can see that the initial value $\psi_{Q1}^{(1)}$ influences only the accuracy in the first iteration.

The optimization process permits a quite wide range of the initial value $\psi_{Q1}^{(1)}$ for quickly producing the optimal results within a small number of iterations. The assumed deviation of 20° is actually already a quite large deviation assumption if emission polarization angle from preliminary numerical simulation are adopted as $\psi_{Q1}^{(1)}$, as the emission polarization is not very sensitive to the change of structure parameters as demonstrated in Supplementary Note 1. Therefore, this optimization process can be quite robust. Even if we start from $\psi_{Q1}^{(1)}$ with a too large deviation (e.g., 60°) from ψ_{Q1}, the optimization can still quickly converge to stable values. However, in this case the optimization target is no longer ψ_{Q1} (for selective detection of Q2) and (θ_1,φ_1) (for selective excitation of Q1), but actually changes to ψ_{Q2} (for selective detection of Q1) and (θ_2,φ_2) (for selective excitation of Q2). In other words, the optimization process shown in Supplementary Fig. 13a actually changes to that in Supplementary Fig. 13b. For example, if $\psi_{Q1}^{(1)}$ is equal to $\psi_{Q1}+60^\circ$, $T_{Q2}\left(\psi_{Q1}^{(1)}+90^\circ\right)$ (34.5%) will be significantly smaller than $T_{Q1}\left(\psi_{Q1}^{(1)}+90^\circ\right)$ (74.5%), and therefore the intended selective detection of Q1 will actually be selective detection of Q2.

Supplementary Note 6 - Exponential fitting of fluorescence decay curves

The spontaneous fluorescence decay curves measured when Q1 is selectively excited (blue solid data points in Fig. 4), when Q2 is selectively excited (red solid data points in Fig. 4), and when Q1 and Q2 are equally excited (yellow-green solid data points in Fig. 4), are fitted using the following bi-exponential decay function

$$y = y_0 + A_1 e^{-(t-t_0)/\tau_1} + A_2 e^{-(t-t_0)/\tau_2}\,. \tag{44}$$

For these three fits, the lifetime parameters τ_1 and τ_2 are shared. The fitting results are as follows. When Q1 is selectively excited (blue solid data points in Fig. 4), the fitting result is

$$y_{\text{Q1-SE}} = 7.4\times10^{-4} + 0.11e^{-(t-10\text{ns})/1.88\text{ns}} + 0.92e^{-(t-10\text{ns})/6.47\text{ns}}\,. \tag{45}$$

When Q2 is selectively excited (red solid data points in Fig. 4), the fitting result is

$$y_{\text{Q2-SE}} = 3.3\times10^{-4} + 1.1e^{-(t-10\text{ns})/1.88\text{ns}} + 0.016e^{-(t-10\text{ns})/6.47\text{ns}} . \quad (46)$$

When Q1 and Q2 are equally excited (yellow-green solid data points in Fig. 4), the fitting result is

$$y_{\text{Q1+Q2}} = 2.8\times10^{-4} + 0.79e^{-(t-10\text{ns})/1.88\text{ns}} + 0.27e^{-(t-10\text{ns})/6.47\text{ns}} . \quad (47)$$

From the fitting results, we can obtain the proportions of the decay components with different lifetimes. The proportion of the photon counts from the decay component with lifetime τ_1 is

$$\frac{A_1\tau_1}{A_1\tau_1 + A_2\tau_2} , \quad (48)$$

while the proportion of the photon counts from the decay component with lifetime τ_2 is

$$\frac{A_2\tau_2}{A_1\tau_1 + A_2\tau_2} . \quad (49)$$

When Q1 is selectively excited, ~96.6% of the photon counts comes from the decay with a lifetime of ~6.47 ns, while only ~3.4% comes from the decay with a lifetime of ~1.88ns. When Q2 is selectively excited, ~95.2% comes from the decay with a lifetime of ~1.88 ns, while only ~4.8% comes from the decay with a lifetime of ~6.47 ns. When Q1 and Q2 are equally excited, ~54% comes from the decay with a lifetime of ~6.47 ns, while ~46% comes from the decay with a lifetime of ~1.88 ns.

In the decay of the selectively excited Q1 (Q2), the minor ~3.4% (~4.8%) decay component with a lifetime of ~1.88 ns (~6.47 ns) makes the decay slightly bi-exponential. Since the lifetime of the minor decay component is equal to the lifetime of the other QD, we attribute the slight bi-exponential nature to the finite excitation selectivity.

The spontaneous fluorescence decay curves measured when Q1 is selectively detected (blue hollowed data points in Fig. 4) and when Q2 is selectively excited (red hollowed data points in Fig. 4) can also be fitted to slightly bi-exponential decay curves with the lifetimes 1.88 ns and 6.47 ns, as follows. When Q1 is selectively detected (blue hollowed data points in Fig. 4), the fitting result is

$$y_{\text{Q1-SD}} = 6.6\times10^{-4} + 0.085e^{-(t-10\text{ns})/1.88\text{ns}} + 0.89e^{-(t-10\text{ns})/6.47\text{ns}} . \quad (50)$$

When Q2 is selectively excited (red hollowed data points in Fig. 4), the fitting result is

$$y_{\text{Q2-SD}} = 2.7\times10^{-4} + 1.1e^{-(t-10\text{ns})/1.88\text{ns}} + 0.012e^{-(t-10\text{ns})/6.47\text{ns}} . \quad (51)$$

When Q1 is selectively detected, ~97.3% of the photon counts comes from the decay with a lifetime of ~6.47 ns, while only ~2.7% comes from the decay with a lifetime of ~1.88ns. When Q2 is selectively detected, ~96.4% comes from the decay with a lifetime of ~1.88 ns, while only ~3.6% comes from the decay with a lifetime of ~6.47 ns. We attribute the slight bi-exponential nature to the finite detection selectivity.

Since the lifetime curves are nearly mono-exponential under selective excitation or selective detection and the minor decay component can be attributed to the other QD due to the finite selectivity, the decay of each QD can be regarded as mono-exponential. When only Q1 is in the nanosystem, the measured lifetime curve is indeed mono-exponential (Supplementary Fig. 9a).

Supplementary Note 7 - Photon collection efficiency

To measure the excitation cross sections or excitation enhancement factors for QDs, the collection efficiency for QD emission is required. Here, the required collection efficiency is a relative efficiency normalized to the collection efficiency for a horizontally oriented linear dipole. The collection efficiency for QD emission is influenced by three factors: dipole orientation, structure loss and optical transmittance.

Before the QD is coupled to the plasmonic nanostructure, the detection efficiency of QD emission is influenced by the orientation of the QD (approximately a 2D-dipole; see Supplementary Fig. 8a), which can be determined by the measured polarization (Fig. 2b) and the numerical aperture of the collective objective (NA = 0.7) [8]. After the QD is coupled to the plasmonic nanostructure, the radiation pattern is tailored to a linear dipole with horizontal orientation (Fig. 2b and Fig. 3b).

Due to the loss in the plasmonic nanostructure the effective quantum efficiency of the nanosystem is less than 100%[9,10].The measured large effective Purcell factors show that nearly all the energy from the exciton decay is extracted to the plasmonic mode excited by a y-oriented dipole (Supplementary Fig. 8d,e). Therefore, the effective quantum efficiency, which is defined as the ratio between the counts of far-field photons and the counts of radiative recombinations of excitons, can be obtained through simulation using a y-oriented dipole. The simulated effective quantum efficiencies for Q1 and Q2 are shown in Supplementary Fig. 8f.

When a linear polarizer is inserted for selective detection, the collection efficiency for the selectively detected QD is further influenced by the transmittance of the polarizer, which can be determined according to the polarizer angle and the measured emission polarization of the selectively detected QD (Fig. 3b).

Supplementary Note 8 - Plasmon-mediated entanglement

Structure of the nanosystem

To make the plasmon-mediated interaction between the emitters much faster than dephasing processes in typical solid-state quantum emitters, we modify the plasmonic nanostructure as shown in Supplementary Fig. 15a. The widths of the gaps are significantly reduced from ~25 nm to 6 nm. The sizes of the three constituent GNRs are also reduced: the diameters are reduced from ~25 nm to 10 nm, while the lengths are reduced from ~86 nm to 42 nm. The two quantum emitters are identical and modelled as two point dipoles $\boldsymbol{\mu}_1$ and $\boldsymbol{\mu}_2$ oscillating along the y-direction with the same transition dipole moment, i.e., $\boldsymbol{\mu}_1 = \mu_1 \mathbf{n}_y$ and $\boldsymbol{\mu}_2 = \mu_2 \mathbf{n}_y$, where $|\mu_1| = |\mu_2| = \mu$. The emitters are positioned 3 nm above the substrate, considering a practical emitter at the center of a nanoparticle with a diameter of 6 nm and placed on the substrate. The parameters s_1 and s_2 (see definition of s_1 and s_2 in Supplementary Fig. 3) are 1.5 nm, which is optimized for selective excitation.

Quantum dynamics

In this nanosystem, the plasmon dissipation is much faster than plasmon-emitter interactions, so the coupling between plasmon and emitters is in the weak-coupling regime. We can trace out the plasmonic degrees of freedom and focus on the dynamics of the reduced density matrix $\hat{\rho}$ for the emitters' subsystem, which is described by the following master equation[11,12]

$$\frac{\partial}{\partial t}\hat{\rho} = -\frac{\mathrm{i}}{\hbar}\left[\hat{H},\hat{\rho}\right] + L(\hat{\rho}), \tag{52}$$

where $\hat{H} = \sum_{i=1,2} \hbar(\omega_{\mathrm{e}} + \delta_i)\hat{\sigma}_i^{\dagger}\hat{\sigma}_i + \sum_{i\neq j}\hbar g_{ij}\hat{\sigma}_i^{\dagger}\hat{\sigma}_j + \sum_i \hbar\Omega_i\left(\hat{\sigma}_i^{\dagger} + \hat{\sigma}_i\right)$ is the effective Hamiltonian of the emitter subsystem, while $L(\hat{\rho}) = -\sum_{i,j}\frac{1}{2}\gamma_{ij}\left(\hat{\sigma}_i^{\dagger}\hat{\sigma}_j\hat{\rho} + \hat{\rho}\hat{\sigma}_i^{\dagger}\hat{\sigma}_j - 2\hat{\sigma}_i\hat{\rho}\hat{\sigma}_j^{\dagger}\right)$ is the Lindblad superoperator. In the first term of $\hat{H}$, ω_{e} is the transition frequency, and δ_i is the lamb shift, which is induced by emitter-plasmon interaction. The second term of $\hat{H}$ describes the coherent interaction between emitters leading to exciton energy level shifts, while $L(\hat{\rho})$ describes the exciton relaxations induced by self and mutual interactions. The third term of $\hat{H}$ describes the resonant excitation on emitter i with a Rabi frequency Ω_i. The key parameters g_{ij} and γ_{ij} in the master equation are the coherent and non-coherent interaction rates, respectively, which can be determined using classical Green's function $\overleftrightarrow{\mathbf{G}}\left(\mathbf{r}_i,\mathbf{r}_j,\omega_{\mathrm{e}}\right)$ as[12,13]

$$g_{ij} = \frac{\omega_{\mathrm{e}}^2}{\hbar\varepsilon_0 c^2}\mathrm{Re}\left[\boldsymbol{\mu}_i^*\overleftrightarrow{\mathbf{G}}\left(\mathbf{r}_i,\mathbf{r}_j,\omega_{\mathrm{e}}\right)\boldsymbol{\mu}_j\right], \tag{53}$$

$$\gamma_{ij} = \frac{2\omega_{\mathrm{e}}^2}{\hbar\varepsilon_0 c^2}\mathrm{Im}\left[\boldsymbol{\mu}_i^*\overleftrightarrow{\mathbf{G}}\left(\mathbf{r}_i,\mathbf{r}_j,\omega_{\mathrm{e}}\right)\boldsymbol{\mu}_j\right]. \tag{54}$$

Since the transition dipoles of the quantum emitters in the nanosystem are set to the y-direction, i.e., $\boldsymbol{\mu}_1 = \mu_1\mathbf{n}_y$ and $\boldsymbol{\mu}_2 = \mu_2\mathbf{n}_y$, only the component $G_{\mathrm{yy}}\left(\mathbf{r}_i,\mathbf{r}_j,\omega_{\mathrm{e}}\right)$ of the Green's function needs to be determined, according to the relationship

$$G_{\mathrm{yy}}\left(\mathbf{r}_i,\mathbf{r}_j,\omega_{\mathrm{e}}\right) = \frac{\varepsilon_0 c^2}{\omega_{\mathrm{e}}^2\mu_j}E_y\left(\mathbf{r}_i,\mathbf{r}_j,\omega_{\mathrm{e}}\right), \tag{55}$$

where $E_{\mathrm{y}}\left(\mathbf{r}_i,\mathbf{r}_j,\omega_{\mathrm{e}}\right)$ is the y-component electric field at $\mathbf{r}_i$ when an electric dipole source $\mu_j\mathbf{n}_y$ is placed at $\mathbf{r}_j$, which is numerically calculated using FDTD simulation.

The simulated g_{ij} and γ_{ij} versus transition wavelength are shown in Supplementary Fig. 15b, where all the rate parameters are in units of the free-space spontaneous decay rate γ_0, which can be expressed as $\gamma_0 = \frac{2\omega_{\mathrm{e}}^2}{\hbar\varepsilon_0 c^2}\mu^2\,\mathrm{Im}\left[\overleftrightarrow{\mathbf{G}}_0(\mathbf{r}_0,\mathbf{r}_0,\omega_{\mathrm{e}})\right]$, where $\overleftrightarrow{\mathbf{G}}_0(\mathbf{r}_0,\mathbf{r}_0,\omega_{\mathrm{e}})$ is the vacuum Green's function, ω_{e} is the transition frequency. Due to structural symmetry, we have $g_{12} = g_{21}$, $\gamma_{12} = \gamma_{21}$, $\gamma_{11} = \gamma_{22} = \gamma$. At the resonance wavelength 808 nm, the interaction is purely dissipative, with a vanishing g_{12} and maximal γ and γ_{12}. The extremely enhanced dissipative interaction γ_{12} is ~72,000 times faster than the intrinsic decay rate γ_0 of the emitters. For a typical transition dipole moment of 10 Debye, the free-space decay rate γ_0 is ~(16.8 ns)$^{-1}$ at ~808 nm transition wavelength, and the enhanced dissipative interaction γ_{12} reaches ~(0.23 ps)$^{-1}$. Such an interaction rate can feasibly overwhelm dephasing processes in typical solid-state quantum emitters[14-18].

For ideally identical emitters or emitters with finite but small transition energy difference in realistic implementations, as long as the plasmon-enhanced interaction rates are much larger than the dephasing rates, the eigenstates of the singly excited emitter subsystem are the maximally entangled states $|\pm\rangle = \left(1/\sqrt{2}\right)\left(|e_1,g_2\rangle \pm |g_1,e_2\rangle\right)$, where $|g_i\rangle$ ($|e_i\rangle$) denotes the ground (excited) state of the emitter $\boldsymbol{\mu}_i$[5]. Using the basis consisting of the singly excited states $|\pm\rangle$, the ground state $|0\rangle = |g_1,g_2\rangle$ and the doubly

excited state $|2\rangle = |e_1, e_2\rangle$, the master equation (Supplementary Equation 52) can be solved and gives an energy shift of $\pm g_{12}$ for state $|\pm\rangle$ and the following rate equations for the diagonal matrix elements of the reduced density matrix $\hat{\rho}$ [13]

$$\begin{aligned} \dot{\rho}_{00} &= (\gamma + \gamma_{12})\rho_{++} + (\gamma - \gamma_{12})\rho_{--}, \\ \dot{\rho}_{++} &= -(\gamma + \gamma_{12})\rho_{++} + (\gamma + \gamma_{12})\rho_{22}, \\ \dot{\rho}_{--} &= -(\gamma - \gamma_{12})\rho_{--} + (\gamma - \gamma_{12})\rho_{22}, \\ \dot{\rho}_{22} &= -2\gamma\rho_{22}. \end{aligned} \tag{56}$$

The diagonal matrix elements determine the population probabilities of the corresponding states: $p_{|0\rangle} = \rho_{00}$, $p_{|+\rangle} = \rho_{++}$, $p_{|-\rangle} = \rho_{--}$, $p_{|2\rangle} = \rho_{22}$. The energy shifts and population dynamics are schematically depicted in Supplementary Fig. 15c. From the rate equations (Supplementary Equation 56), we can see that the decay rate of the symmetric state $|+\rangle$ is $\gamma_+ = \gamma + \gamma_{12}$ (red curve in Supplementary Fig. 15d), while that of the anti-symmetric state $|-\rangle$ is $\gamma_- = \gamma - \gamma_{12}$ (blue curve in Supplementary Fig. 15d). Since γ_{12} is positive and close to γ around the plasmonic resonance wavelength (~808 nm), the symmetric state $|+\rangle$ is super-radiant while the anti-symmetric state $|-\rangle$ is sub-radiant. The super-radiant state $|+\rangle$ decays at rate $\gamma_+ \sim 1.48\times10^5\gamma_0$, which is ~40 times faster than the decay rate of the sub-radiant state $|-\rangle$ ($\gamma_- \sim 3.74\times10^3\gamma_0$). As we demonstrate below, this large decay rate difference leads to spontaneous generation of entanglement between the emitters.

Spontaneous entanglement generation

As an example demonstration of spontaneous entanglement generation, the system is initialized to a singly excited unentangled state $|e_1, g_2\rangle$ (a superposition of states $|+\rangle$ and $|-\rangle$: $|e_1, g_2\rangle = \left(1/\sqrt{2}\right)\left(|+\rangle + |-\rangle\right)$). This initial state can be prepared by selective excitation of emitter $\boldsymbol{\mu}_1$ as shown in Supplementary Fig. 16. Due to the height mismatch between the emitter and the GNRs, there are significant z-components of the local fields at the emitters, which reduce the theoretical excitation selectivity. Nevertheless, under such an unfavourable condition, the excitation selectivity is still sufficiently high for experiment.

In the basis $\{|0\rangle, |+\rangle, |-\rangle, |2\rangle\}$, the density matrix of the initial state $|e_1, g_2\rangle$ is expressed as

$$\hat{\rho}(0) = \begin{pmatrix} 0 & 0 & 0 & 0 \\ 0 & 1/2 & 1/2 & 0 \\ 0 & 1/2 & 1/2 & 0 \\ 0 & 0 & 0 & 0 \end{pmatrix}. \tag{57}$$

Since the system is initialized in a singly excited state and left to decay spontaneously without driving, the dynamics is confined in the reduced basis $\{|0\rangle, |+\rangle, |-\rangle\}$, and the rate equations for the population evolutions can be reduced to

$$\begin{aligned} \dot{\rho}_0 &= (\gamma + \gamma_{12})\rho_{++} + (\gamma - \gamma_{12})\rho_{--}, \\ \dot{\rho}_{++} &= -(\gamma + \gamma_{12})\rho_{++}, \\ \dot{\rho}_{--} &= -(\gamma - \gamma_{12})\rho_{--}, \end{aligned} \tag{58}$$

from which the spontaneous evolutions of the populations are obtained as

$$\begin{aligned} \rho_0 &= 1 - \tfrac{1}{2}e^{-(\gamma+\gamma_{12})t} - \tfrac{1}{2}e^{-(\gamma-\gamma_{12})t}, \\ \rho_{++} &= \tfrac{1}{2}e^{-(\gamma+\gamma_{12})t}, \\ \rho_{--} &= \tfrac{1}{2}e^{-(\gamma-\gamma_{12})t}, \end{aligned} \tag{59}$$

which are plotted in Supplementary Fig. 15e. The entanglement is evaluated using concurrence $C = \sqrt{(\rho_{++} - \rho_{--})^2 + 4\,\mathrm{Im}(\rho_{+-})^2}$ [12], where the matrix element $\rho_{+-} = \tfrac{1}{2}e^{-\gamma t}e^{-i(2g_{12}t)}$, which is obtained

through solving rate equation $\dot{\rho}_{+-} = -(\gamma + 2g_{12}i)\rho_{+-}$ with the initial condition $\rho_{+-}(0) = \frac{1}{2}$. Around the resonance wavelength 808nm, g_{12} is vanishing, therefore ρ_{+-} is real and the concurrence is reduced to $C = \rho_{++} - \rho_{--}$ (solid curve in Supplementary Fig. 15e). From Supplementary Fig. 15e, we can see that both states $|\pm\rangle$ decays from a population probability of 0.5. The state $|+\rangle$ decays very quickly while the state $|-\rangle$ decays much more slowly, which quickly leads to imbalance between states $|\pm\rangle$ and thus induces entanglement. At $t = \ln\left[(\gamma + \gamma_{12})/(\gamma - \gamma_{12})\right]/(2\gamma_{12}) = 2.5\times10^{-5}\tau_0$, the entanglement reaches a maximum $C = 0.44$, where the population of the fast decaying state $|+\rangle$ become negligible as compared with the slow decaying state $|-\rangle$. After that, the entanglement decays slowly at approximately the decay rate of state $|-\rangle$ until the dephasing processes (not explicitly involved in the master equation and the rate equations) destroy the entanglement. For wavelength slightly detuned from the plasmonic resonance 808 nm, the non-vanishing g_{12} will induce a slight oscillation in the time evolution of the concurrence.

Entanglement detection

The states $|\pm\rangle$ decay to different plasmonic modes as shown by the electric field profiles in Supplementary Fig. 15f,g. The field profiles are numerically calculated by coherently adding the field profiles from dipole sources $\boldsymbol{\mu}_1$ and $\boldsymbol{\mu}_2$ with phase delay 180° for state $|+\rangle$ and 0° for state $|-\rangle$. The plasmons from the decay of state $|+\rangle$ radiate to x-polarized photons with radiation efficiency of ~1.9% (red curve in Supplementary Fig. 15h), while the plasmons from the decay of state $|-\rangle$ radiate to y-polarized photons with radiation efficiency of ~9.3% (blue curve in Supplementary Fig. 15h). Therefore, through polarization analysis of the photon radiation, states $|\pm\rangle$ can be distinguished and the state of the system can be analyzed.

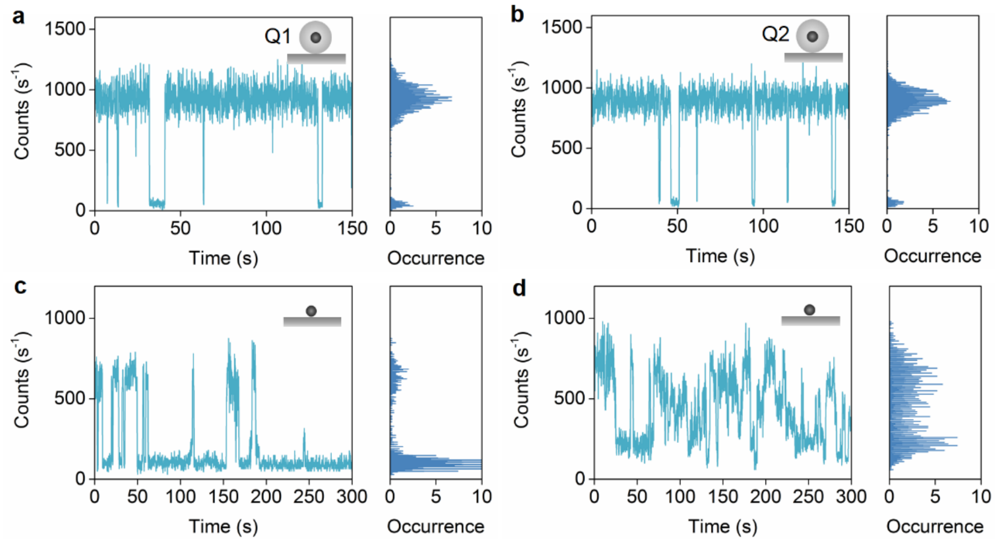

Supplementary Figure 1 Emission stability of single QDs on the substrate. **a**,**b** The emission intensity time trajectories (left) and the corresponding distributions of intensity (right) for Q1 (**a**) and Q2 (**b**), which are encapsulated with a silica shell. QDs with silica-encapsulation blink only occasionally and have stable bright-state intensity level. **c**,**d** The emission intensity time trajectories (left) and the corresponding histograms of the distribution of intensities (right) for two typical QDs without silica-encapsulation. QDs without silica-encapsulation blink frequently and part of them have rather broad distributions of intensity (e.g., in panel **d**).

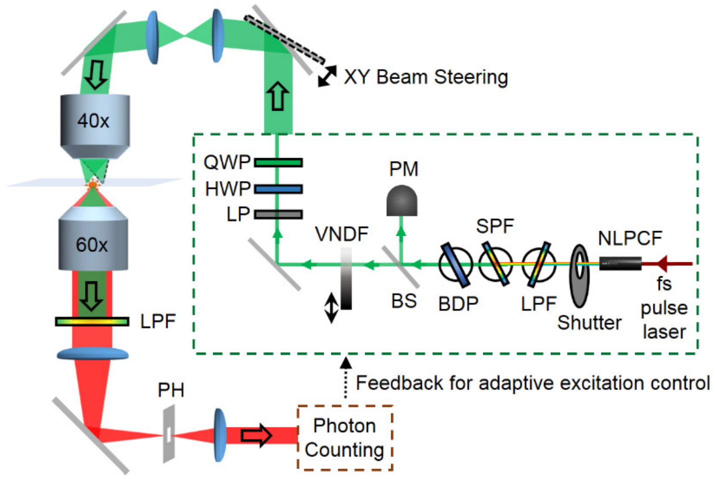

Supplementary Figure 2 Optical setup for photoluminescence characterization. The optical system is a home-built microscopic single-molecule fluorescence detection system, consisting of an excitation module, a microscope, and a photon counting module. NLPCF, nonlinear photonic crystal fiber; LPF, long-pass edge filter; SPF, short-pass edge filter; BDP, beam displacement plate; BS, beam splitter; PM, power meter; VNDF, variable neutral density filter; LP, linear polarizer; HWP, half-waveplate; QWP, quarter-waveplate; PH, pinhole.

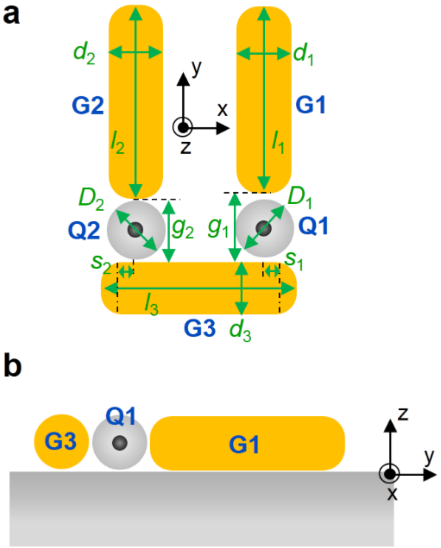

Supplementary Figure 3 Structure parameters of the nanosystem. **a** View from +z direction. The parameters l_1, l_2 and l_3 are the lengths of G1, G2 and G3, respectively. The parameters d_1, d_2 and d_3 are the diameters of G1, G2 and G3, respectively. The parameters D_1 and D_2 are the diameters of Q1 and Q2 (including the silica shell), respectively. The parameters g_1 and g_2 are the gap widths between G1 and G3 and between G2 and G3, respectively. The end-caps of the GNRs are modelled as half oblate spheroids with an aspect ratio of 0.5. The parameter s_1 (s_2) is the x-directional distance from the center of G1 (G2) to the equatorial plane of the spheroidal end-cap of G3. **b** View from +x direction.

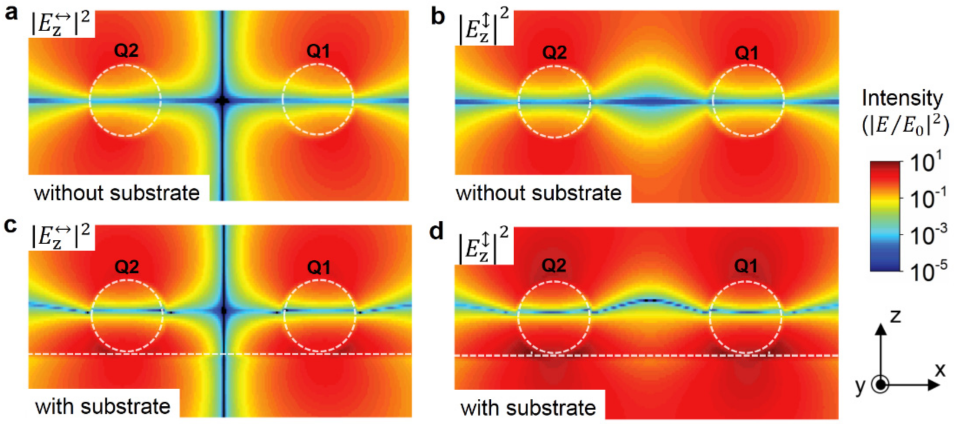

Supplementary Figure 4 Simulated z-component electric field distributions in the xz plane passing through the center of the QDs when excited with x- and y-polarized light at 740 nm wavelength. **a** Source-normalized intensities of the z-component electric fields when there is no substrate and when excited with x-polarized light (denoted as ↔). **b** Same as panel (**a**) but when excited with y-polarized light (denoted as ↕). **c**,**d** Same as panels (**a**) and (**b**) but when there is the substrate. The intensity distributions in (**a**)-(**d**) share the colour bar.

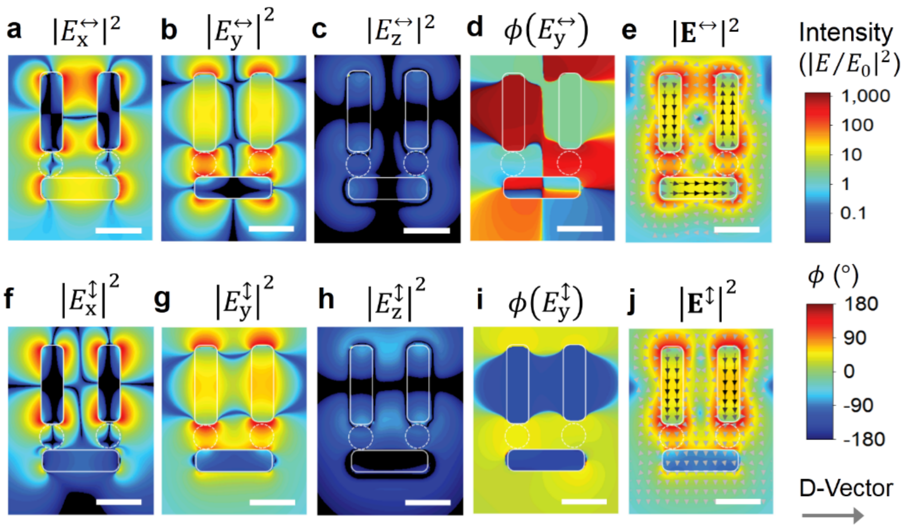

Supplementary Figure 5 Numerically simulated field distributions when excited with x- and y-polarized light at 740 nm wavelength. **a**-**c** Distributions of the source-normalized intensities of the x- (**a**), y- (**b**) and z- (**c**) component electric fields when excited with x-polarized light (denoted as ↔). **d** Distribution of the phases of the y-component electric fields when excited with x-polarized light (denoted as ↔). **e** Distribution of the source-normalized intensities of the electric fields and the arrow plots of the electric displacement vectors when excited with x-polarized light (denoted as ↔). **f**-**h** Distributions of the source-normalized intensities of the x- (**f**), y- (**g**) and z- (**h**) component electric fields when excited with y-polarized light (denoted as ↕). **i** Distribution of the phases of the y-component electric fields when excited with y-polarized light (denoted as ↕). **j** Distribution of the source-normalized intensities of the electric fields and the arrow plots of the electric displacement vectors when excited with y-polarized light (denoted as ↕). Scale bars in (**a**)-(**j**) are 50 nm. The intensity distributions in (**a**)-(**c**), (**e**), (**f**)-(**h**) and (**j**) share the colour bar for electric field intensity. The phase distributions in (**d**) and (**i**) share the colour bar for phase.

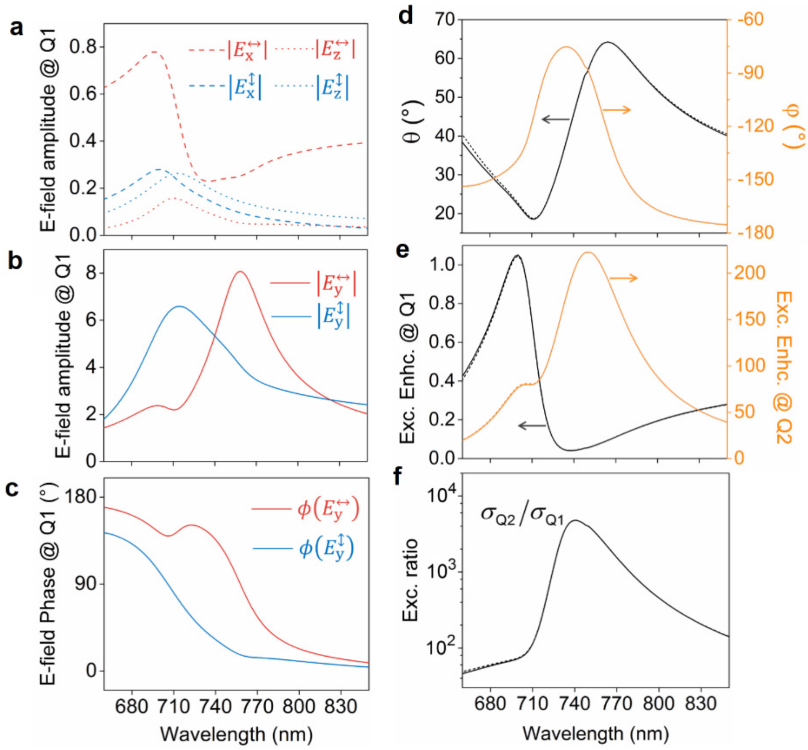

Supplementary Figure 6 Excitation suppression of Q1 (selective excitation of Q2) in a broad range of wavelength. **a** Source-normalized amplitude spectra of the x- (dashed curves) and z- (dotted curves) component electric field at Q1 when excited with x- (red curves) and y- (blue curves) polarized light. **b** Source-normalized amplitude spectra of the y-component electric field at Q1 when excited with x- (red curve) and y- (blue curve) polarized light. **c** Phase spectra of the y-component electric field at Q1 when excited with x- (red curve) and y- (blue curve) polarized light. **d** Polarization parameters (θ, φ) for optimal excitation suppression of Q1 (selective excitation of Q2) at different wavelengths. The solid curves are determined through direct calculation using equations $\theta = \arctan\left(\left|E_y^{\leftrightarrow}\right| / \left|E_y^{\updownarrow}\right|\right)$ and $\varphi = \phi\left(E_y^{\leftrightarrow}\right) - \phi\left(E_y^{\updownarrow}\right) - 180^\circ$ described in Supplementary Note 2. The dashed curves are determined using the searching method described in Supplementary Note 5. **e** Excitation enhancement factors for Q1 (left y-axis) and Q2 (right y-axis) when excited with optimal polarization parameters (θ, φ) at different wavelengths (shown in panel **d**). **f** Excitation ratio σ_{Q2}/σ_{Q1} when excited with optimal polarization parameters (θ, φ) at different wavelengths (shown in panel **d**).

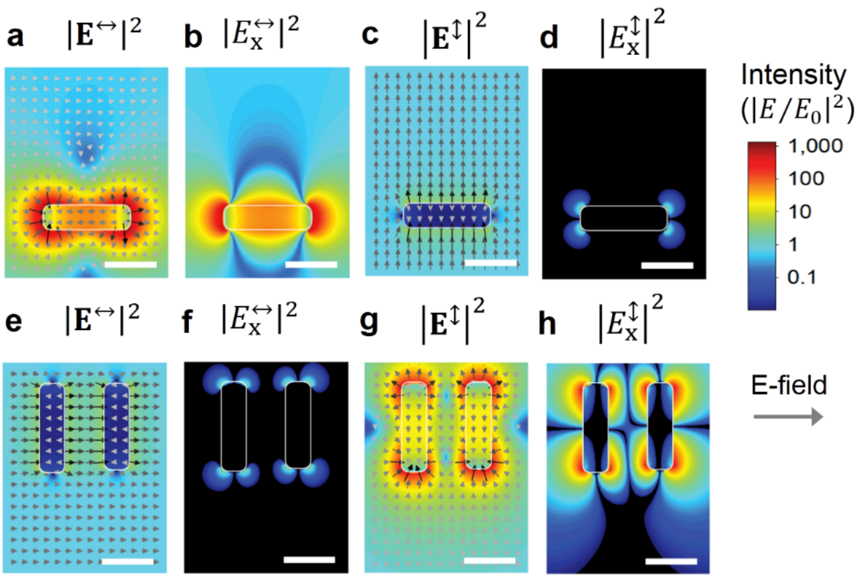

Supplementary Figure 7 Field distributions simulated separately for two parts of the U-shaped nanostructure when excited with x- and y-polarized light at 740 nm wavelength. **a**,**b** Source-normalized intensities and arrow plots of the electric fields (**a**) and source-normalized intensities of the x-component electric fields (**b**) when excited with x-polarized light (denoted as ↔) for the part composed of G3. **c**,**d** Same as panels (**a**) and (**b**) but excited with y-polarized light (denoted as ↕). **e**-**h** Same as panels (**a**)-(**d**) but for the part composed of G1 and G2. Scale bars in (**a**)-(**h**) are 50 nm. The intensity distributions in (**a**)-(**h**) share the colour bar.

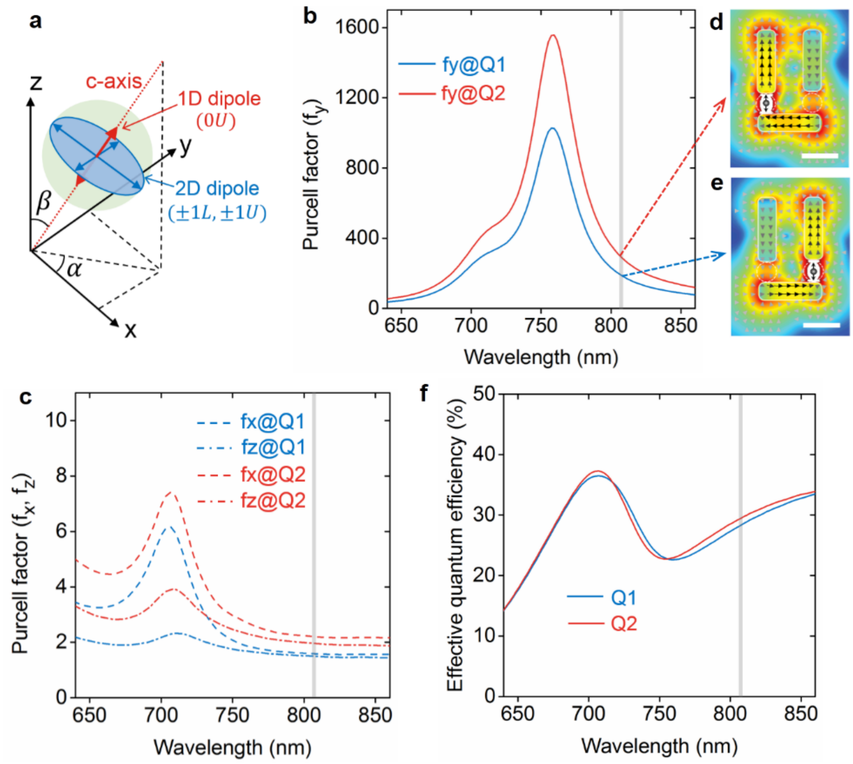

Supplementary Figure 8 Simulation of Purcell effects for the quantum dots in the plasmonic nanostructure. **a** Schematic illustration of the 3D orientation of a spherical QD. **b** Simulated Purcell factor f_y (for y-oriented dipole transitions) at Q1 and Q2. **c** Simulated Purcell factors f_x and f_z (for x- and z-oriented dipole transitions) at Q1 and Q2. **d**,**e** Simulated distributions of electric field intensities and electric displacement vectors of the plasmonic mode excited at 808 nm wavelength by a y-oriented dipole at Q1 (**e**) and Q2 (**d**). Scale bars are 50 nm. **f** Simulated effective quantum efficiencies of Q1 and Q2 in the nanosystem. The grey vertical lines in panels (**b**), (**c**) and (**f**) denote the central emission wavelength (~808 nm) of the QDs.

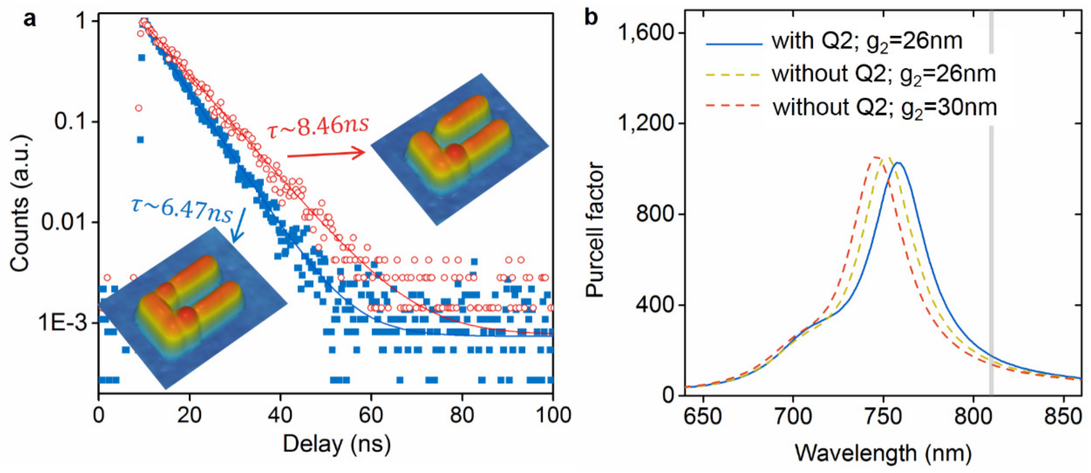

Supplementary Figure 9 Lifetime of Q1 before and after moving Q2 into the nanosystem. **a** Measured lifetime of Q1 before (red hollowed data points and red fitting curve) and after (blue solid data points and blue fitting curve) moving Q2 into the nanosystem. **b** Simulated Purcell factors f_y (for y-oriented dipole transitions) for different structure parameters showing the influence of the refractive index of the silica-encapsulated Q2 (modelled as a silica sphere) and the change of gap width g_2. The grey vertical line denotes the emission wavelength of ~808 nm.

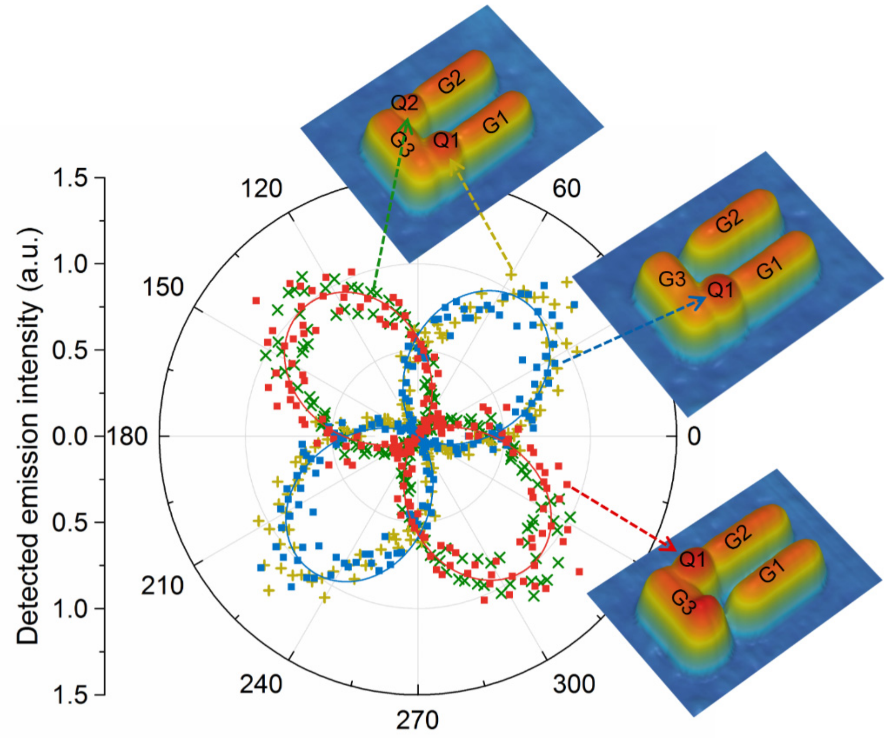

Supplementary Figure 10 Emission polarization of Q1 at different locations of the nanosystem. Polar plots of the emission polarization measurements when Q1 is at the gap between G1 and G3 (blue experimental data points and simulated continuous curve; middle inset) and when Q1 is at the gap between G2 and G3 (red experimental data points and simulated continuous curve; lower inset, the tiny bump on G3 is a fragment that sticks during the manipulation). For comparison, the emission polarizations of selectively excited Q1 (yellow '+'-shaped data points) and Q2 (green 'x'-shaped data points) when both QDs are in the nanosystem are also plotted, which is the same as in Fig. 3b.

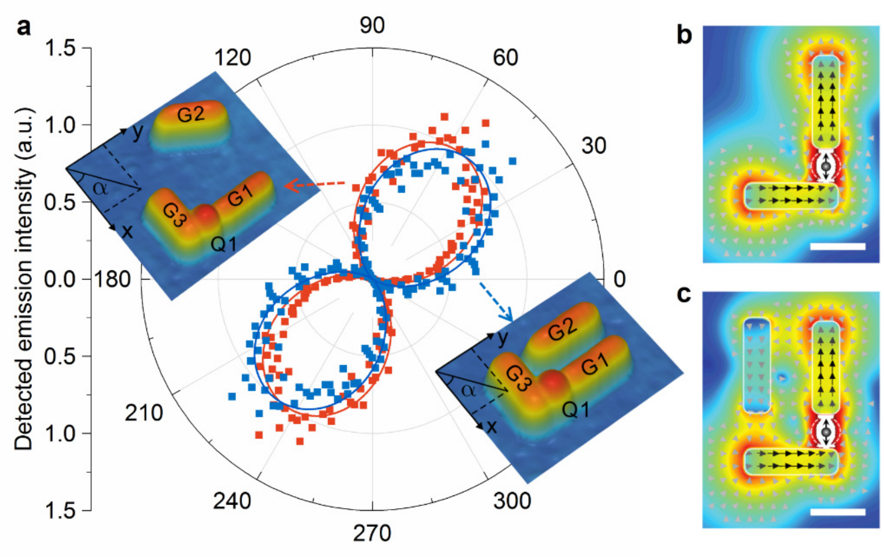

Supplementary Figure 11 Influence of the gold nanorod on the far-field emission polarization. **a** Polar plots of the emission polarization measurements when Q1 is coupled to the L-shaped (before G2 is added) gold nanostructure (upper inset, red experimental data points and simulated continuous curve) and the U-shaped (after G2 is added) gold nanostructure (lower inset, blue experimental data points and simulated continuous curve). **b**,**c** Numerically simulated maps of electric field intensities and electric displacement vectors of the plasmonic mode excited at 808 nm wavelength by a y-oriented dipole located at Q1 in the L- (**b**) and U- (**c**) shaped gold nanostructure.

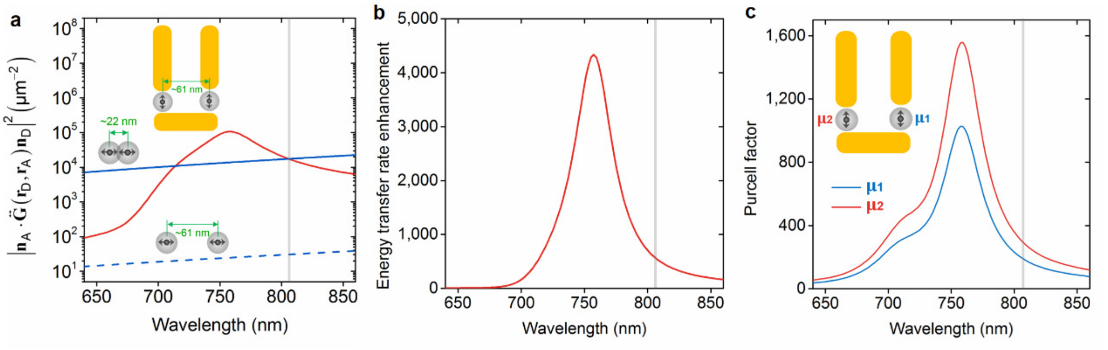

Supplementary Figure 12 Simulated energy transfer enhancement and Purcell effect for dipole emitters in the gold nanostructure. **a** Numerically simulated value of $\left|\mathbf{n}_{\mathrm{A}} \cdot \ddot{\mathbf{G}}(\mathbf{r}_{\mathrm{D}}, \mathbf{r}_{\mathrm{A}})\mathbf{n}_{\mathrm{D}}\right|^2$ (as a function of the wavelength of energy transfer) for three different cases: dipole emitters coupled with the gold nanostructure (red solid curve); dipole emitters ~22 nm apart and without the gold nanostructure (blue solid curve); dipole emitters ~61 nm apart and without the gold nanostructure (blue dashed curve). **b** Enhancement factor of the energy transfer rate between the dipole emitters coupled with the gold nanostructure. **c** Spontaneous emission rate enhancement factors (Purcell factors) for the dipole emitters in the gold nanostructure. The grey vertical lines in panels (**a**)-(**c**) denote the emission wavelength of ~808 nm.

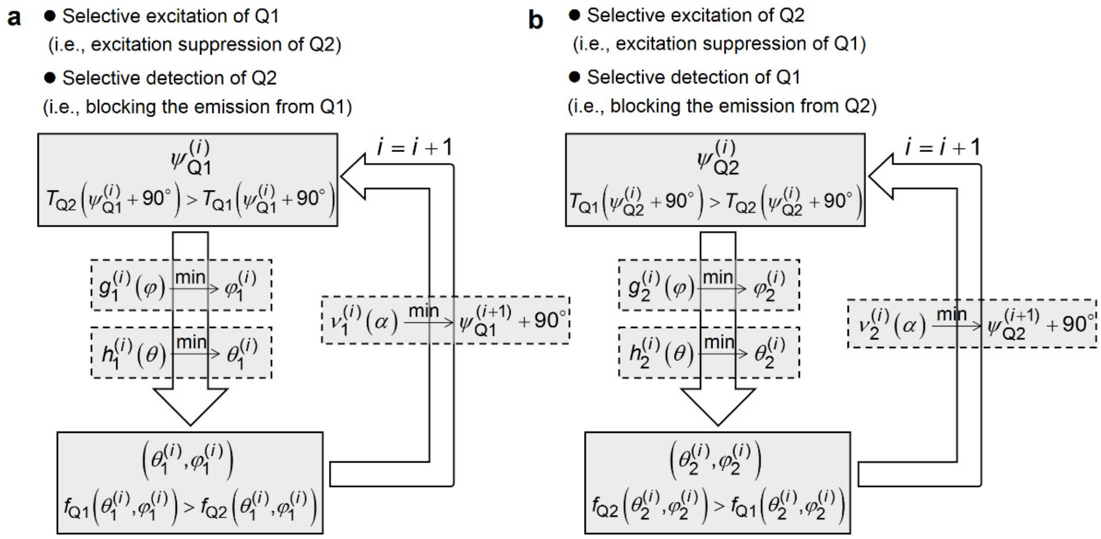

Supplementary Figure 13 Optimization process to get parameters for selective excitation and selective detection. **a** Optimization process for selective excitation of Q1 (i.e., excitation suppression of Q2) and selective detection of Q2 (i.e., blocking the emission from Q1). **b** Optimization process for selective excitation of Q2 (i.e., excitation suppression of Q1) and selective detection of Q1 (i.e., blocking the emission from Q2).

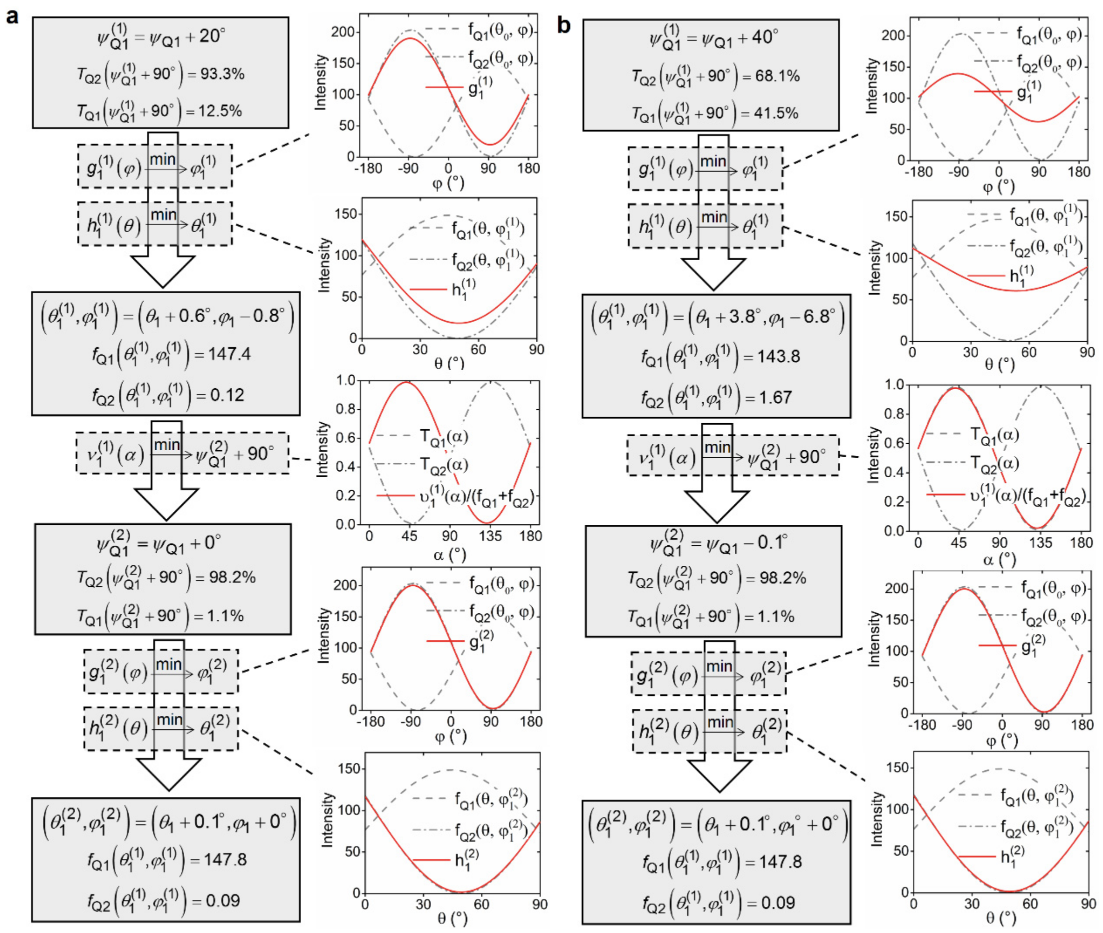

Supplementary Figure 14 Theoretical demonstration of the optimization process for the structure simulated in the main text. **a** Demonstration of the optimization process that start with a $\psi_{Q1}^{(1)}$ equal to $\psi_{Q1} + 20^\circ$. **b** Demonstration of the optimization process that start with a $\psi_{Q1}^{(1)}$ equal to $\psi_{Q1} + 40^\circ$.

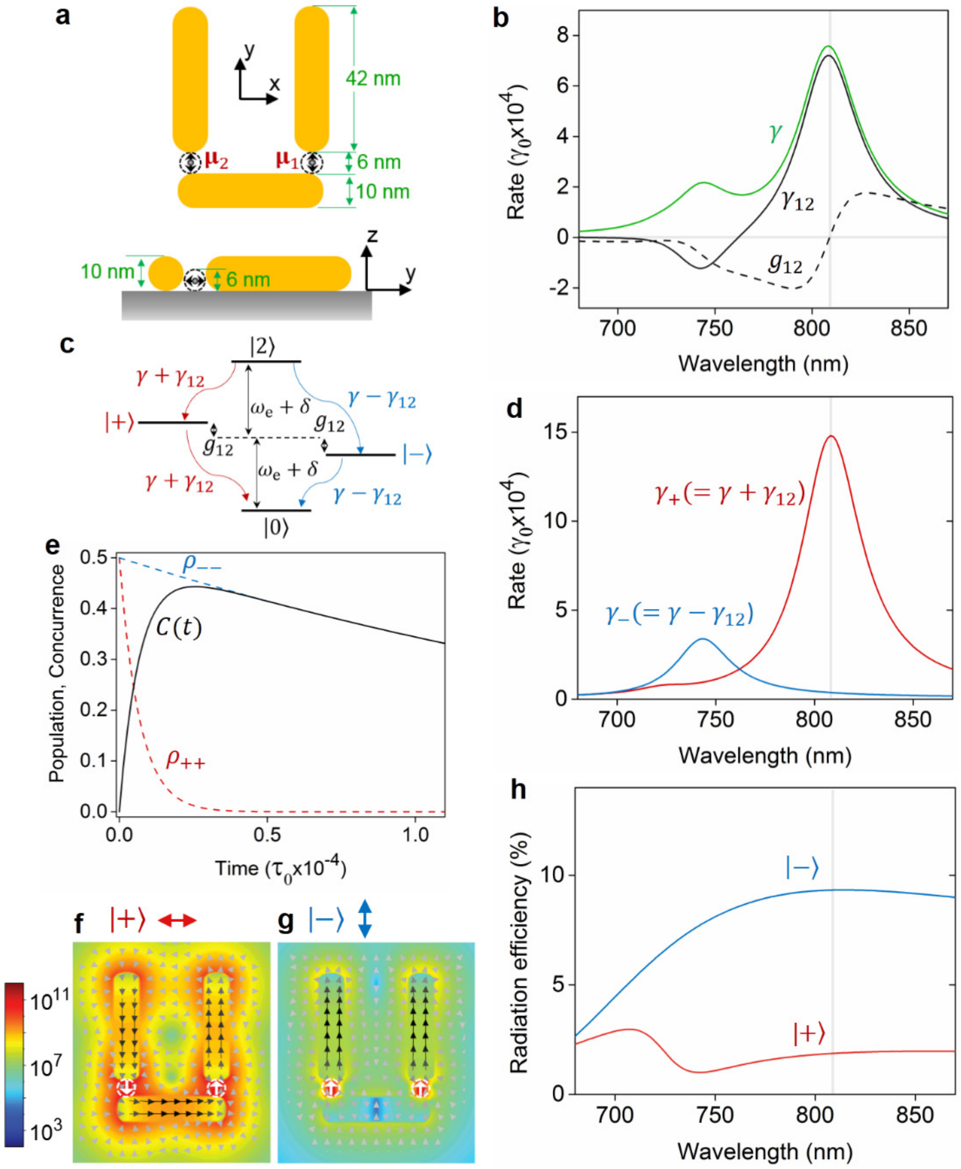

Supplementary Figure 15 Plasmon-mediated entanglement between the emitters in the modified nanosystem. **a** Structure parameters of the nanosystem. The three GNRs are identical. The two quantum emitters are identical and modelled as two point dipoles $\boldsymbol{\mu}_1$ and $\boldsymbol{\mu}_2$ oscillating along the y-direction with the same transition dipole moment. **b** Simulated rate parameters for the emitters' subsystem versus transition wavelength. The vertical grey line denotes the wavelength 808 nm, where $g_{12} = 0$ while γ_{12} is at its peak value. **c** Schematic of the level structure and decay channels. **d** Simulated decay rate of states $|\pm\rangle$ versus transition wavelength. The vertical grey line denotes the wavelength 808 nm, where the decay rate of state $|+\rangle$ reaches its maximum. **e** Spontaneous evolution of the population probabilities of states $|\pm\rangle$ ($\rho_{\pm\pm}$) and the concurrence (*C*), at 808 nm wavelength, where $g_{12} = 0$. **f**,**g** Distributions of electric field intensities and electric displacement vectors of the plasmonic modes from the decay of state $|+\rangle$ (**f**) and $|-\rangle$ (**g**) at 808 nm wavelength. The double arrow on top of the profile indicates the corresponding polarization of the far-field radiation. The two profiles share the colour bar. **h** Far-field radiation efficiency (the fraction of energy radiated into the far field) for states $|\pm\rangle$. The vertical grey line denotes the wavelength 808 nm.

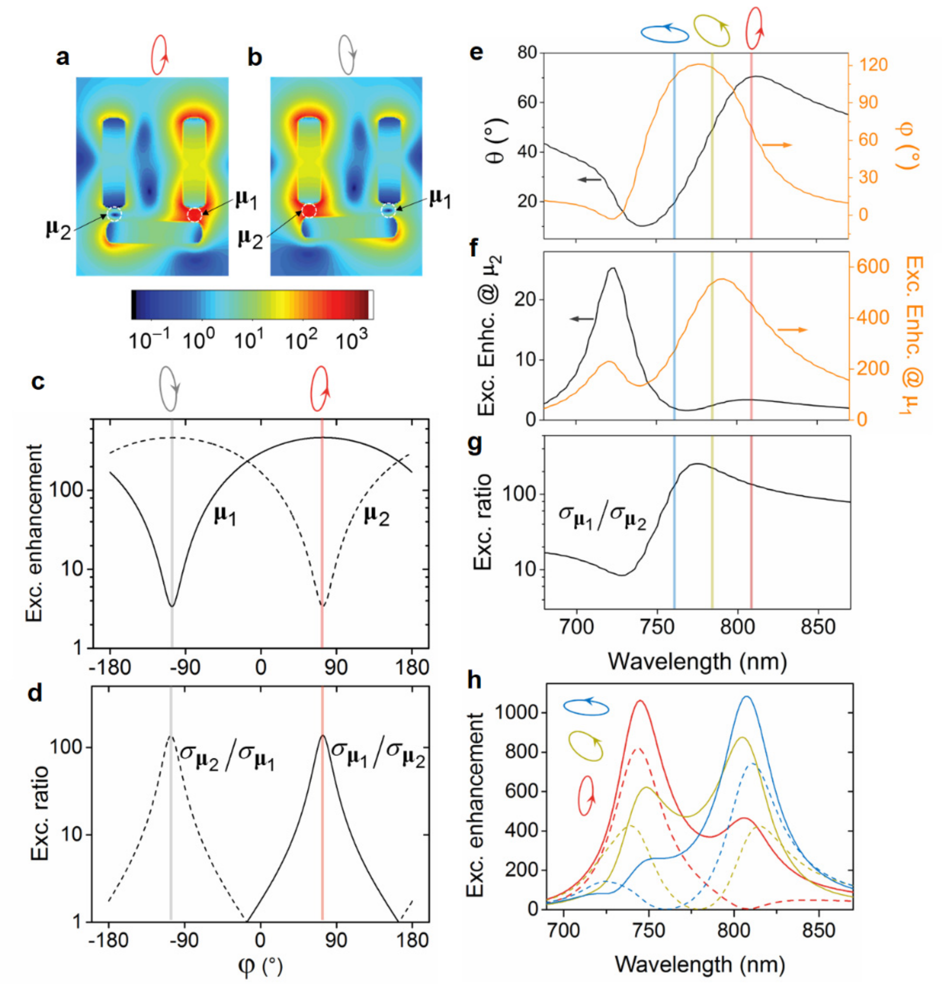

Supplementary Figure 16 Selective excitation of the emitters in the modified nanosystem for entanglement study. **a**,**b** Distribution of source-normalized electric field intensity when μ_1 (**a**) or μ_2 (**b**) is selectively excited at 808 nm wavelength using the elliptical polarization displayed on the top of the panel. The optimal excitation polarization parameters $(\theta,\ \varphi)$ are (70°, 73.6°) (**a**) and (70°, -106.5°) (**b**), respectively. The profile plane is 5 nm above the substrate. **c** Excitation enhancement factors for μ_1 (solid curve) and μ_2 (dashed curve) as φ varies (θ is 70°; excitation wavelength is 808 nm). **d** Excitation ratio $\sigma_{\mu_1}/\sigma_{\mu_2}$ (solid curve) and $\sigma_{\mu_2}/\sigma_{\mu_1}$ (dashed curve) as φ varies (θ is 70°; excitation wavelength is 808 nm). **e** Polarization parameters $(\theta,\ \varphi)$ for optimal selective excitation of μ_1 (optimal excitation suppression of μ_2) at different wavelengths. The optimal polarization ellipses are displayed on the top of the panel for excitation wavelengths 760 nm, 780 nm and 808 nm. **f** Excitation enhancement factors for μ_2 (left y-axis) and μ_1 (right y-axis) when the system is excited with optimal polarization parameters $(\theta,\ \varphi)$ at different wavelengths (shown in panel **e**). **g** Excitation ratio $\sigma_{\mu_1}/\sigma_{\mu_2}$ when the system is excited with optimal polarization parameters $(\theta,\ \varphi)$ at different wavelengths (shown in panel **e**). **h** Excitation enhancement spectra for μ_1 (solid curves) and μ_2 (dashed curves) when the system is excited using elliptical polarizations for optimal selective excitation of μ_1 at 760 nm (blue curves), 780 nm (yellow-green curves) and 808 nm (red curves).

Supplementary Table 1 Structure parameter modifications and their influences on selective excitation and emission polarization.

	Structure parameter modification	Selective excitation of Q1 (excitation @ 740 nm)		Selective excitation of Q2 (excitation @740 nm)		emission polarization of Q1 (@ 808 nm)			emission polarization of Q2 (@ 808 nm)		
		$(\theta,\ \varphi)$ (*)	$\frac{\sigma_{Q1}}{\sigma_{Q2}}$ (*)	$(\theta,\ \varphi)$ (*)	$\frac{\sigma_{Q2}}{\sigma_{Q1}}$ (*)	ψ_{Q1} (*)	χ_{Q1} (*)	DOLP(*)	ψ_{Q2} (*)	χ_{Q2} (*)	DOLP(*)
#0	-	(49.2, 93.7)	1560	(49.2, -86.3)	1560	43.6	-4.5	0.988	136.4	4.5	0.988
#1	l_1: +3 nm	(31.9, 93.4)	1720	(47.8, -97.6)	1100	47	-3.5	0.993	139.3	5.9	0.979
#2	l_1: -3 nm	(61.4, 71.8)	896	(50.2, -79.2)	2070	40.5	-5.4	0.982	134.3	3.7	0.992
#3	d_1: +1 nm	(54.9, 90.3)	1270	(48.8, -83.4)	1530	42.7	-4.8	0.986	136	4.3	0.989
#4	d_1: -1 nm	(31.2, 90.4)	1510	(47.5, -94.7)	1420	45.7	-3.9	0.991	138.6	5.7	0.980
#5	d_3: +1 nm	(48.5, 88.8)	1420	(48.5, -91.2)	1420	45.5	-4.2	0.989	134.5	4.2	0.989
#6	d_3: -1 nm	(46.9, 107.5)	1640	(46.9, -72.5)	1640	38.3	-6.2	0.977	141.7	6.2	0.977
#7	g_1: +5 nm	(56.6, 67.6)	644	(48.7, -88)	1440	45.8	-4.5	0.988	131.5	3.2	0.994
#8	s_1: -6 nm	(47.9, 93)	1370	(51.5, -91.5)	490	42.9	-4.2	0.989	136.6	4.6	0.987
#9	s_1: +6 nm	(47.8, 93.1)	1620	(44, -80.4)	661	47.1	-4.9	0.985	135.8	4.5	0.988
#10	d_{x1}: +4 nm	(49.2, 93.7)	1460	(49.8, -86.8)	450	42.8	-4.6	0.987	136.4	4.5	0.988
#11	d_{x1}: -4 nm	(49.2, 93.7)	1430	(48.3, -85.9)	539	44.6	-4.5	0.988	136.4	4.5	0.988
#12	d_{y1}: +4 nm	(49.2, 93.7)	2330	(45.9, -80.8)	2490	50.8	-4.8	0.986	136.4	4.5	0.988
#13	d_{y1}: -4 nm	(49.2, 93.7)	1490	(53.9, -92.4)	1090	35.7	-4	0.990	136.4	4.5	0.988
#14	d_{z1}: +4 nm	(49.2, 93.7)	1420	(49.2, -85.9)	1530	43.8	-4.4	0.988	136.4	4.5	0.988
#15	d_{z1}: -4 nm	(49.2, 93.7)	1420	(49, -86.6)	170	43.8	-4.7	0.987	136.4	4.5	0.988

(*) $(\theta,\ \varphi)$: the excitation polarization parameters; σ_{Q1}/σ_{Q2} : excitation ratio; ψ_{Q1} (ψ_{Q2}): the angle of the major axis of the emission polarization ellipse of Q1 (Q2); χ_{Q1} (χ_{Q2}): the arc tangent of the ratio between the semi-minor and semi-major diameters of the emission polarization ellipse of Q1 (Q2); DOLP: the degree of linear polarization.

Supplementary References

1. Le Thomas, N., Herz, E., Schops, O., Woggon, U. & Artemyev, M. V. Exciton fine structure in single CdSe nanorods. *Physical Review Letters* **94**, 016803 (2005).
2. Empedocles, S. A., Neuhauser, R. & Bawendi, M. G. Three-dimensional orientation measurements of symmetric single chromophores using polarization microscopy. *Nature* **399**, 126-130 (1999).
3. Labeau, O., Tamarat, P. & Lounis, B. Temperature dependence of the luminescence lifetime of single CdSe/ZnS quantum dots. *Physical Review Letters* **90**, 257404 (2003).
4. Efros, A. L., Rosen, M., Kuno, M., Nirmal, M., Norris, D. J. & Bawendi, M. Band-edge exciton in quantum dots of semiconductors with a degenerate valence band: Dark and bright exciton states. *Physical Review B* **54**, 4843-4856 (1996).
5. Novotny, L. & Hecht, B. *Principles of Nano-Optics*. (Cambridge University Press, New York, 2006).
6. Bidault, S., Devilez, A., Ghenuche, P., Stout, B., Bonod, N. & Wenger, J. Competition between forster resonance energy transfer and donor photodynamics in plasmonic dimer nanoantennas. *ACS Photonics* **3**, 895-903 (2016).
7. Wu, P. G. & Brand, L. Resonance energy-transfer - methods and applications. *Analytical Biochemistry* **218**, 1-13 (1994).
8. Lethiec, C., Laverdant, J., Vallon, H., Javaux, C., Dubertret, B., Frigerio, J. M., Schwob, C., Coolen, L. & Maitre, A. Measurement of three-dimensional dipole orientation of a single fluorescent nanoemitter by emission polarization analysis. *Physical Review X* **4**, 021037 (2014).
9. Kinkhabwala, A., Yu, Z. F., Fan, S. H., Avlasevich, Y., Mullen, K. & Moerner, W. E. Large single-molecule fluorescence enhancements produced by a bowtie nanoantenna. *Nature Photonics* **3**, 654-657 (2009).
10. Akselrod, G. M., Argyropoulos, C., Hoang, T. B., Ciraci, C., Fang, C., Huang, J. N., Smith, D. R. & Mikkelsen, M. H. Probing the mechanisms of large Purcell enhancement in plasmonic nanoantennas. *Nature Photonics* **8**, 835-840 (2014).
11. He, Y. & Zhu, K. D. Strong coupling among semiconductor quantum dots induced by a metal nanoparticle. *Nanoscale Research Letters* **7**, 95 (2012).
12. Gonzalez-Tudela, A., Martin-Cano, D., Moreno, E., Martin-Moreno, L., Tejedor, C. & Garcia-Vidal, F. J. Entanglement of two qubits mediated by one-dimensional plasmonic waveguides. *Physical Review Letters* **106**, 020501 (2011).
13. Martín-Cano, D., González-Tudela, A., Martín-Moreno, L., García-Vidal, F. J., Tejedor, C. & Moreno, E. Dissipation-driven generation of two-qubit entanglement mediated by plasmonic waveguides. *Physical Review B* **84**, 235306 (2011).
14. Batalov, A., Zierl, C., Gaebel, T., Neumann, P., Chan, I. Y., Balasubramanian, G., Hemmer, P. R., Jelezko, F. & Wrachtrup, J. Temporal coherence of photons emitted by single nitrogen-vacancy defect centers in diamond using optical Rabi-oscillations. *Physical Review Letters* **100**, 077401 (2008).
15. Rogers, L. J., Jahnke, K. D., Teraji, T., Marseglia, L., Muller, C., Naydenov, B., Schauffert, H., Kranz, C., Isoya, J., McGuinness, L. P. & Jelezko, F. Multiple intrinsically identical single-photon emitters in the solid state. *Nature Communications* **5**, 4739 (2014).
16. Kuhlmann, A. V., Prechtel, J. H., Houel, J., Ludwig, A., Reuter, D., Wieck, A. D. & Warburton, R. J. Transform-limited single photons from a single quantum dot. *Nature Communications* **6**, 8204 (2015).
17. Empedocles, S. A., Neuhauser, R., Shimizu, K. & Bawendi, M. G. Photoluminescence from single semiconductor nanostructures. *Advanced Materials* **11**, 1243-1256 (1999).
18. Bonadeo, N. H., Erland, J., Gammon, D., Park, D., Katzer, D. S. & Steel, D. G. Coherent optical control of the quantum state of a single quantum dot. *Science* **282**, 1473-1476 (1998).
